# Bivalent chromatin accommodates survivin and BRG1/SWI complex to activate DNA damage response in CD4^+^ cells

**DOI:** 10.1186/s12964-024-01814-4

**Published:** 2024-09-11

**Authors:** Venkataragavan Chandrasekaran, Karin M. E. Andersson, Malin Erlandsson, Shuxiang Li, Torbjörn Nur Olsson, Maria-Jose Garcia-Bonete, Eric Malmhäll-Bah, Pegah Johansson, Gergely Katona, Maria I. Bokarewa

**Affiliations:** 1 Department of Rheumatology and Inflammation Research, Institute of Medicine, Sahlgrenska Academy, University of Gothenburg, Box 480, Gothenburg, 40530 Sweden; 2 Rheumatology Clinic, Sahlgrenska University Hospital, Gröna Stråket 16, Gothenburg, 41346 Sweden; 3 Computational Biology and Biophysics Lab, Queen’s University, Kingston, Canada; 4 Department of Chemistry and Molecular Biology, Faculty of Science, University of Gothenburg, Gothenburg, Sweden; 5 Department of Medical Biochemistry and Cell Biology, Institute of Biomedicine, University of Gothenburg, Gothenburg, SE 405 30 Sweden; 6 Department of Clinical Chemistry, Sahlgrenska University Hospital, Gothenburg, Sweden

**Keywords:** Bivalent chromatin, DNA damage, BRG1, Survivin, Autoimmunity

## Abstract

**Background:**

Bivalent regions of chromatin (BvCR) are characterized by trimethylated lysine 4 (H3K4me3) and lysine 27 on histone H3 (H3K27me3) deposition which aid gene expression control during cell differentiation. The role of BvCR in post-transcriptional DNA damage response remains unidentified. Oncoprotein survivin binds chromatin and mediates IFNγ effects in CD4^+^ cells. In this study, we explored the role of BvCR in DNA damage response of autoimmune CD4^+^ cells in rheumatoid arthritis (RA).

**Methods:**

We performed deep sequencing of the chromatin bound to survivin, H3K4me3, H3K27me3, and H3K27ac, in human CD4^+^ cells and identified BvCR, which possessed all three histone H3 modifications. Protein partners of survivin on chromatin were predicted by integration of motif enrichment analysis, computational machine-learning, and structural modeling, and validated experimentally by mass spectrometry and peptide binding array. Survivin-dependent change in BvCR and transcription of genes controlled by the BvCR was studied in CD4^+^ cells treated with survivin inhibitor, which revealed survivin-dependent biological processes. Finally, the survivin-dependent processes were mapped to the transcriptome of CD4^+^ cells in blood and in synovial tissue of RA patients and the effect of modern immunomodulating drugs on these processes was explored.

**Results:**

We identified that BvCR dominated by H3K4me3 (H3K4me3-BvCR) accommodated survivin within *cis*-regulatory elements of the genes controlling DNA damage. Inhibition of survivin or JAK-STAT signaling enhanced H3K4me3-BvCR dominance, which improved DNA damage recognition and arrested cell cycle progression in cultured CD4^+^ cells. Specifically, BvCR accommodating survivin aided sequence-specific anchoring of the BRG1/SWI chromatin-remodeling complex coordinating DNA damage response. Mapping survivin interactome to BRG1/SWI complex demonstrated interaction of survivin with the subunits anchoring the complex to chromatin. Co-expression of BRG1, survivin and IFNγ in CD4^+^ cells rendered complete deregulation of DNA damage response in RA. Such cells possessed strong ability of homing to RA joints. Immunomodulating drugs inhibited the anchoring subunits of BRG1/SWI complex, which affected arthritogenic profile of CD4^+^ cells.

**Conclusions:**

BvCR execute DNA damage control to maintain genome fidelity in IFN-activated CD4^+^ cells. Survivin anchors the BRG1/SWI complex to BvCR to repress DNA damage response. These results offer a platform for therapeutic interventions targeting survivin and BRG1/SWI complex in autoimmunity.

**Supplementary Information:**

The online version contains supplementary material available at 10.1186/s12964-024-01814-4.

## Background

Bivalent chromatin regions (BvCR) are defined by concomitant deposition of histone H3 tails modified by trimethylation of lysine residues at position 4 (H3K4me3) and 27 (H3K27me3) and have a wide distribution across the genome [1, 2]. It is well-studied in developmental biology wherein, spatiotemporal control of developmental stages is achieved by resolution of the BvCR into active, dominated by H3K4me3, or poised, dominated by H3K27me3 regions [1]. Mathematical modelling [3, 4] and experimental evidence [5] indicates duality of BvCR where co-existence of both modifications empowers finetuning of gene transcription. In terminally differentiated cells, retention of bivalency maintains flexibility of transcription in response to environmental stimuli. BvCR play an important role in lineage commitment of CD4^+^T cells [5–8], and B cells [9]. However, their precise function in terminally differentiated immunocompetent cells is still debated.

Occupancy of BvCR modifications in H3 tails is mediated by chromatin remodeling complexes including BRG1-associated SWItch/Sucrose Non-Fermentable (BRG1/SWI) complex, Polycomb Repressive Complex 2 (PRC2), and Complex of Proteins Associated with Set1 (COMPASS). The BRG1/SWI complex utilizes energy from ATP-dependent hydrolyses to translocate along DNA promoting chromatin accessibility for transcriptional regulation, and to disrupt the DNA-histone contact for post-transcriptional remodeling [10]. Nucleosome binding and remodeling activity of BRG1/SWI complex is seriously affected by histone modifications. For example, polyacetylation of H3 promotes stable chromatin association, while H3K4me3 involved in DNA damage recognition has been reported to inhibit BRG1/SWI activity [11, 12].

Transcription is considered a natural source of genome instability [13, 14]. Thus, the necessity of post-transcriptional repair of DNA lesions evolved well-organized pathways of DNA damage sensing, signal transducers of stress activating factors, and the damage repair machinery intimately connected with chromatin packaging, cell cycle control and apoptotic responses [15]. Histone modifications preserve genome integrity [12, 16, 17]. For example, phosphorylation of histone H2AX at serine residue 139 (γH2AX) occurs rapidly at DNA lesions and serves a platform for recruitment of DNA repair complexes. Histone methylation abundance triggers DNA damage responses and contributes to DNA repair [16, 17]. However, the function of chromatin bivalency in DNA damage control remains largely unexplored.

Survivin, encoded by the *BIRC5* gene, is best known as part of the Chromosomal Passenger Complex [18, 19] and for its role in cell cycle control through interaction with histone H3 tail [20, 21]. Cytoplasmic localization of survivin leads to anti-apoptotic activity [22], while nuclear survivin acts in gene transcription through association with transcription factors [23–25]. Survivin is important for the maturation of lymphocytes [26–30]. Survivin deletion in thymocytes prevents formation of functional T cell receptor and obstructs T cell development [27, 29]. In mature CD4^+^ cells, survivin is essential for maintaining the effector activity of T cells contributing to IFNγ-signaling and autoimmune inflammation [25, 28].

Given that survivin interacts with threonine-3 residue of histone H3 [20, 21], and with the catalytic subunit of the PRC2 complex preventing trimethylation of histone H3K27 [31], it prompted us to investigate if survivin binds to the BvCR containing H3K4me3 and H3K27me3, and if such binding affected the function of CD4^+^ cells. We report that intersect of chromatin sequences within BvCR and survivin delineate their targeted colocalization within *cis*-regulatory elements of the CD4^+^ genome. Subsequently, sequence-specific annotation to the transcription factors’ landscape predict enrichment of the BRG1/SWI complex subunits within those BvCR. This prediction was consolidated by the chromatin immunoprecipitated by survivin, composition-based machine learning, protein docking approach, and a peptide binding assay, all of which convergently exposed survivin and BRG1/SWI complex interaction in proximity of the nucleosome. Further, functional studies were performed to explore survivin dependence of BvCR involvement in the DNA damage response mediated by the BRG1/SWI complex. Finally, we postulate the clinical role of survivin-BRG1/SWI complex axis in pathogenic CD4^+^ cells abundant in synovia of patients with rheumatoid arthritis, and the ability of anti-rheumatic drugs to modulate the DNA damage response.

## Methods

### Human material

Blood samples of 67 RA patients and 40 healthy controls, all collected at the Rheumatology Clinic, Sahlgrenska Hospital, Gothenburg, were used in this study. Clinical characteristics of the patients are shown in Supporting Figure S1A.

### Isolation and stimulation of CD4^+^ cells

Human mononuclear cells were isolated from the venous peripheral blood by density gradient separation on Lymphoprep (Axis-Shield PoC As, Dundee, Scotland). CD4^+^ cells were isolated by positive selection (Invitrogen, 11331D), and cultured at density 1.25 × 10^6^ cells/ml in wells coated with anti-CD3 antibody (0.5 μg/ml; OKT3, Sigma-Aldrich, St.Louis, Missouri, USA), in RPMI medium (Gibco, Waltham, Massachusetts, USA) supplemented with 50 μM β-mercaptoethanol (Gibco), Glutamax 2 mM (Gibco), gentamicin 50 μg/ml (Sanofi-Aventis, Paris, France) and 10% fetal bovine serum (Sigma-Aldrich) at 37 °C in a humidified 5% CO_2_ atmosphere. For RNA-seq, CD4^+^ cell cultures were treated with recombinant IFNγ (50 ng/ml; Peprotech, Cranbury, NJ, USA) and survivin inhibitor sepantronium bromide (YM155 [32]) (10 nM; Selleck Chemicals, Houston, TX) for 72 h. For qPCR, CD4^+^ cells were treated with IFNγ, YM155, or JAK-inhibitor tofacitinib (10 µM; Selleck Chemicals) for 48 h.

### DNA content and cell cycle analysis

Freshly isolated PBMC from 16 healthy persons were seeded 10^6^/ml in flat bottom wells, stimulated for 48 h with anti-CD3 antibodies with tofacitinib (0 or 10 µM) or YM155 (0, 1, 5, 10 or 25 nM). Eight cultures were additionally treated with recombinant IFNγ (0 or 50 ng/ml) for the last 18 h. At harvest, cells were first blocked with Fc-block (BD 564220), stained with AF647-conjugated antibodies to CD4^+^ (Biolegend 317,422) followed by permeabilization (eBioscience, 00–5521-00, ThermoFisher), and incubated overnight, 4 °C, with 20 μg/ml of 7-aminoactinomycin D (7AAD, Invitrogen A1310) in perm/wash (BD Biosciences), and resuspended in 200μL FACS buffer.

In addition, the human monocytic cell line THP-1 (TIB-202, ATCC, Manassas, VA, USA) propagated in RPMI1649 medium supplemented with 10% FCS, 50 μM β-mercaptoethanol (Gibco), 1 mM sodium pyruvate, 10 mM HEPES, and gentamycin in a humidified atmosphere of 5% CO 2 at 37 °C. For the experiments, expanding cells were seeded 0.3 × 10^6^/ml in flat-bottom wells and stimulated for 96 h with YM155 (0, 1, 5, 10, and 25 nM). For proliferation experiments cells were prestained before culture with CellTrace Violet Proliferation dye (CTV, ThermoFischer) according to the manufacturer’s instructions. For cell cycle analysis cells were permeabilized, and incubated overnight, 4 °C, with 7AAD in perm/wash (BD Biosciences) and resuspended in FACS buffer.

Cells were acquired with flow cytometry system BD FACSLyric™ (BD Biosciences) and data analysis was performed in the Tree Star FlowJo software using the inbuilt cell cycle analysis tool, the wizard auto gate compensation and Watson model with constraints, CV (G2) = CV (G1) and the proliferation wizard tool set for 4 peaks.

### Quantitative (q)PCR

RNA was isolated with the Total RNA Purification Kit (#17,200, Norgen Biotek). RNA concentration and quality were evaluated with a NanoDrop spectrophotometer (ThermoFisher Scientific) and Experion electrophoresis system (Bio-Rad Laboratories). cDNA was synthesized from RNA (400 ng) with the High-Capacity cDNA Reverse Transcription Kit (Applied Biosystems, Foster City, CA, USA). Real-time amplification was done with RT2 SYBR Green qPCR Mastermix (Qiagen) and a ViiA 7 Real-Time PCR System (ThermoFisher Scientific) as described [33]. Primers are shown in Supporting Figure S1B.

### Primer design

Primers were designed by the Primer3 web client (https://primer3.ut.ee/). When applicable, primers were separated by an exon-exon boundary. Amplicon and primer size was limited to 60–150 and 18–24 base pairs, respectively. Melting temperature was set between 60–63 °C, max poly-X to 3 and GC-content was limited to 40–60%. Primers were checked for possible hairpin and primers-dimer structures in Net Primer web (https://www.premierbiosoft.com/netprimer/). Binding of primers was validated in UCSC In-Silico PCR web (http://genome.ucsc.edu/cgi-bin/hgPcr) against the GRCh38/hg38 human genome.

### Transcriptional sequencing (RNA-seq)

RNA of CD4^+^ cells was prepared using the Norgen Total RNA kit (17,200 Norgen Biotek, Ontario, Canada). Quality control was done by Bioanalyzer RNA6000 Pico on Agilent2100 (Agilent, Santa Clara, CA, USA). Deep sequencing was done by RNA-seq (Hiseq2000, Illumina) at the core facility for Bioinformatics and Expression Analysis (Karolinska Institute, Huddinge, Sweden). Raw sequence data were obtained in Bcl-files and converted into fastq text format using the bcl2fastq program from Illumina.

### Chromatin immunoprecipitation and sequencing (ChIP-seq)

For survivin-ChIP-seq analysis, twelve CD4^+^ cell cultures were stimulated with concanavalin A (ConA, 0.625 μg/ml, MP Biomedicals), and lipopolysaccharide (LPS, 5 μg/ml, Sigma-Aldrich) for 72 h. For histone-ChIP-seq analysis, three CD4^+^ cell cultures were stimulated with ConA and LPS as above for 24 h and then treated with YM155 (0 or 10 ng/ml) for 24 h. The cells were cross-linked and lysed with the EpiTect ChIP OneDay kit (Qiagen 334,471). After sonication, cellular debris was removed, and DNA material was pooled. After preclearing, 1% of the sample was saved as an input fraction and used as background binding. Pre-cleared chromatin was incubated with 2 μg of anti-survivin (10,811, Santa Cruz Biotechnology, Santa Cruz, CA, USA), anti-H3K27ac (C15410196, Diagenode), or anti-H3K27me3 (C15410195, Diagenode) or anti-H3K4me3 (C15410003, Diagenode). The immune complexes were washed, the cross-links were reversed, and the DNA was purified with the EpiTect ChIP OneDay kit (Qiagen) as recommended. The quality of purified DNA was assessed with TapeStation (Agilent, Santa Clara, CA, USA). DNA libraries were prepared with ThruPLEX (Rubicon) and sequenced with a Hiseq2000 sequencing system (Illumina). Bcl-files were converted and demultiplexed to fastq with bcl2fastq (Illumina).

### Immunohistochemistry and imaging

Human monocytic cell line THP1 (TIB-202, ATCC, Manassas, VA, USA) were seeded 10^6^/ml on glass chamber slides (Thermo Scientific) precoated with poly-L-lysine (Sigma-Aldrich, Saint Louis, MO, USA). Cells were treated with 10 µM or 50 µM tofacitinib or 200 nM YM155 (both from Selleck chemicals) for 24 h. At harvest, cells were fixed with 4% buffer and formalin for 10 min and blocked and permeabilised for 3 h with 3% normal goat serum and 1% TritonX100. Primary antibodies against H2A.X phosphorylated at Ser^139^ (γH2AX, mouse, Millipore 05–636), BRG1 (rabbit, Bethyl Laboratories A300-813A), H3K4me3 (rabbit, C15410003, Diagenode), and PE-conjugated survivin (mouse, Clone 91,630, RnD systems) and isotype controls were diluted in blocking buffer and the slides were incubated over night at 4 °C. This was followed by Alexa-fluor conjugated secondary antibodies donkey-anti-mouse AF488 (Invitrogen A-21202), goat-anti-rabbit AF488 (Invitrogen A11034) or donkey-anti-rabbit AF647 (Invitrogen A-31573) for 2 h at room temperature. Autofluorescence was blocked with 0.5% Sudan Black B (Sigma-Aldrich) in 70% ethanol for 20 min at room temperature. Nuclei were stained with Hoechst 34,580 (NucBlue Live Cell Stain; Thermo Fisher Scientific) for 20 min and mounted with ProLong Gold antifading mounting reagent (Invitrogen).

### Confocal imaging and analysis

Fluoresence microscopy was performed using the confocal imaging system Leica SP8 (Leica Microsystems, Wetzlar, Germany) with sequencial acquisition using a 40 × oil objective and up to 10 × digital zoom.

The images were aquired at high resolution (1.5 × digital zoom) viewing 40–70 nuclei per image. Within each sample, γH2AX-positive foci were enumerated in 2–3 images resulting in 78–222 nuclei per treatment. Images were analysed with ImageJ version 2.9 within 8-bit composite images [34]. Threshold was adjusted for each antibody to optimise identification of positive spots. Nuclear area was defined by thresholding the Hoechsts (blue) image and exporting the results to ROI. Co-localization of survivin and BRG1 was measured using the JACoP plugin [35]. Number of γH2AX-positive foci in each nuclei stained with Hoechsts was estimated after noise reduction by despeckling using the ImageJ feature Find Maxima.

### Affinity immunoprecipitation

THP-1 cells were lysed in modified RIPA-buffer (25 mM Tris–HCl pH 7.4, 200 mM NaCl, 1 mM EDTA, 1% NonidetP-40, 5% glycerol) supplemented with protease inhibitors (Complete mini, Roche), and immunoprecipitation was performed with 2 mg of anti-survivin antibodies (RnD AF886) and of control rabbit IgG (011–000-002, Jackson ImmunoResearch) coupled to the Dynabeads Protein G Immunoprecipitation Kit (10007D, ThermoFisher Scientific), cross-linked with bis(sulfosuccinimidyl)suberate (A39266, Pierce™). The immuno-precipitated (IP) complexes were washed extensively with the provided washing buffer plus fragment stream buffer containing 0.1% SDS (10 mM Tris–HCl pH 7.5; 2 mM EDTA; 0.1% Triton X-100; 0.1% SDS). Electrophoresis was performed by loading 30 mg of total nuclear extract, and the IP material on NuPage 4–12% Bis–Tris gels (Novex). Protein bands were stained with Coomassie Blue.

### Sample preparation for mass ppectrometry

IP material was digested from electrophoresis gel bands obtained from the pull-down assays. Selected gel bands were dissected and prepared using in-gel digestion protocol [36–38]. Gel pieces were destained in 50% acetonitrile and reduced with 10 mM DTT at 37 °C for 30 min. Samples were alkylated with 25 mM Iodoacetoamide for 20 min at RT protected from light. The gel pieces were washed in acetonitrile between steps. Digestion was performed using 10 ng/μl trypsin (Promega) in 50 mM ammonium bicarbonate, pH 8.0 with overnight incubation at 37 °C. The peptides were extracted with a 66% acetonitrile with 0.2% formic acid solution and speed-vac to remove the organic solvents. Samples were acidified with 5% acetic acid before C18 stage-tip purification [39]. The peptides were resolved in 0.2% formic acid for MS analysis.

### Mass spectrometry and data analysis

MS analysis was performed by LC–MS/MS using a nano HPLC system (EASY-nLC, Thermo Scientific, Odense, Denmark) coupled to a Q-Exactive HF mass spectrometer (ThermoFisher Scientific). Peptides were separated using in-house packed columns (150 × 0.0075 mm) packed with Reprosil-Pur C18-AQ 3 μm particles (Dr. Maisch, Ammerbuch, Germany). Peptide were separated with 5 to 35% gradient (A 0.1% formic acid, B 0.1% formic acid, 80% Acetonitrile) in 30 min. In brief, full mass spectra were acquired over a mass range of minimum 400 m/z and maximum 1600 m/z, with a resolution of at least 60,000 at 200 m/z. The 12 most intense peaks with a charge state ≥ 2—5 were fragmented with normalized collision energy of 27%, and tandem MS was acquired at a resolution of 17,500 and subsequent excluded for selection for 10 s. Proteins peptides were identified using MaxQuant (v1.5.7.4) in a searched against the human proteome from UniProt protein database including 75,400 protein sequence entries. The modifications were set as carbamidomethylation of cysteine (fixed) and oxidation of methionine and protein N-terminal (variable). MS summary data are deposited at the Proteome Xchange database (http://www.ebi.ac.uk/pride) with identifier PXD049683.

### Survivin binding peptide microarray

The peptide microarray experiment was described previously [31]. Briefly, peptide microarray was designed which contained the BRG1/SWI complex subunits using PEPperCHIP Peptide Microarrays (PEPperPRINT Gmbh). Each protein sequence was split into 15 amino acid peptide units, with 10 amino acid overlap, which resulted in 262 peptides representing the BRG1 (P51532) protein, 459 peptides for ARID1A (O14497), 86 peptides for ACTL6A (O96019), 245 peptides for SMARCC2 (Q8TAQ2), 104 peptides SMARCD1 (Q96GM5), 83 peptides for SMARCE1 (Q969G3), 78 peptides for SMARCB1 (Q12824), 79 peptides for DPF2 (Q92785), and 101 peptide PHF10 (Q8WUB8) proteins. In the microarray plate, the peptides were printed in duplicate spots and were framed by additional HA (YPYDVPDYAG, 232 spots) control peptides. Background interactions was examined by pre-staining one microarray with the secondary 6X His Tag Antibody DyLight680 antibody (1:1000) and monoclonal anti-HA (12CA5)-DyLight800 control antibody (1:1000). Another peptide microarray was incubated with recombinant human survivin at a concentration of 1 and 10 μg/ml and stained with the secondary 6X His Tag Antibody DyLight680 antibody (Rockland Immunochemicals, Pottstown, PA, USA) and the monoclonal anti-HA (12CA5)-DyLight800 control antibody (Rockland Immunochemicals, Pottstown, PA, USA). The read-out was performed using LI-COR Odyssey Imaging System with scanning intensities of 7/7 (red/green). HA and His-tag peptides were also stained simultaneously in the assay as internal quality control. PepSlide Analyzer was used for quantification of spot intensities and peptide annotation based on the 16-bit gray scale tiff files. A software algorithm breaks down fluorescence intensities of each spot into raw, foreground and background signal and generated the peptide intensity map. The resulting data was stored as a table with information on protein identifier, peptide sequence, and fluorescence intensity. The fluorescence intensity above 30,000 indicated strong, above 10,000 indicated moderate, and above 1000 indicated low binding regions.

### Modeling of survivin binding with the BRG1/SWI complex

To investigate how survivin binds with the SWI complex, we used cryo-electron microscopy (EM) structures of BAF bound to nucleosomes as a basis for our modeling. Specifically, two distinct forms of BAF-nucleosome complexes, namely canonical BAF (cBAF, PDB ID: 6LTJ) [40] and the polybromo-associated BAF (PBAF, PDB ID: 7VDV) [41], were used in this study. For survivin, the X-ray structure of the human survivin-H3 tail complex was used (PDB ID: 3UEF) [21].

Predictions of the binding modes between survivin and the cBAF and PBAF complexes were conducted through the hybrid protein–protein docking software HDOCK [42]. The default parameters were used for the docking calculation, and subsequent docking conformations were ranked based on docking energy scores. The top ten docking conformations, exhibiting the best docking energy scores, were used as the first criterion to identify plausible interaction modes for survivin-cBAF and survivin-PBAF complexes. Then we conducted the modeling of the full H3 tail (residues 1 to 43) using the partial H3 tail conformations from the BAF-nucleosome complex (H3 tail residues 37 to 43) and the survivin-H3 tail complex (H3 tail residues 1 to 5). Modeling of the complete H3 tail was performed by Modeller using orientations and distance restraints from the two partial H3 tail conformations. This constituted the second criterion for determining reasonable interaction modes out of top 10 docking conformations. Since MS analysis unveiled that survivin interacts with components of the SWI complex, including SMARCA2/4, SMARCC1/C2, SMARCD1/D2, SMARCE1, DPF2, and PBRM1. Consequently, we introduced structural contact restraints as the third criterion to refine the selection of reasonable interaction modes between survivin and SWI complex.

### Bioinformatics analysis

#### RNA-seq analysis

Mapping of transcripts was done using Genome UCSC annotation set for hg38 human genome assembly. Analysis was performed using the core Bioconductor packages in R-studio v. 4.3.1. Differentially expressed genes (DEG) between the samples were identified using DESeq2 (v.1.40.2) with Benjamini–Hochberg adjustment for multiple testing.

#### ChIP-seq analysis

The fastq sequencing files were mapped to the human reference genome (hg38) using the STAR aligner [43] with default parameters apart from setting the alignIntronMax flag to 1 for end-to-end mapping. Quality control of the sequenced material was performed by FastQC tool using MultiQC v.0.9dev0 (Babraham Institute, Cambridge, U.K.). Peak calling was performed using the HOMER [44] findPeaks command, with 1 tag per base pair counted (-tbp 1). For peak calling in histone ChIP-seq, the option -style histone was used to find broad regions of enrichment. Peaks were filtered for the histone H3 antibody or survivin antibody IP fraction and unprocessed DNA (Input), which is a generally accepted normalization approach to identify protein-specific enrichment of DNA interaction areas [45]. A set of peaks with enrichment versus surrounding region and Input (adjusted *p* < 10e − 5) was identified and quantified separately for each sample. Peaks were annotated with HOMER software in standard mode to the closest TSS. Peaks with overlapping localization by at least one nucleotide were merged and further on referred to as one peak. To quantify strength of binding and maintain consistency of comparison in the histone H3 samples and survivin sample, peak score was calculated by the position adjusted reads from initial peak region.

#### Tag quantification for ChIP-seq comparison

To quantify ChIP-seq tag densities from different ChIP-seq experiments, the HOMER annotatePeaks command was used, with the following parameters: -size given -noadj -pc 1. Normalized Tag Counts were calculated separately for the histone H3 ChIP-seq peaks and survivin ChIP-seq samples and presented the number of tags found at the peak, normalized to 10 million total mapped tags [46].

#### Identification of BvCR

The R package ChIPpeakAnno [47], version 3.34.1 was used to identify bivalent chromatin regions, using the input ChIP-seq peaks of survivin, and the three histone H3 modifications. The function ‘findOverlapsofPeaks’ was used, with parameters restricting the maximum gap between peak ranges to zero, indicating a minimum of one bp overlap, and connected peak ranges within multiple groups as ‘merged’. The resulting set of BvCR was separated into dominant H3-BvCR. To define dominant BvCR, tags within each H3 mark of the BvCR were summed and the H3 mark that contributed the highest percentage of tags to the BvCR was designated as the dominant H3 mark in that BvCR. Peak scores of survivin within the dominant H3K4me3-BvCR, H3K27me3-BvCR, and H3K27ac-BvCR were analysed. Changeable BvCR were defined as those BvCR that showed a shift in the dominant H3 modification after YM155 treatment, for example, a BvCR that was initially dominant in H3K4me3 prior to YM155 treatment shifting to dominating in H3K27me3 after YM155 treatment, analysed through maximum tag percentage before and after YM155 treatment.

Matrix values to calculate the peak score per BvCR corresponding to H3K4me3, H3K27me3, and H3K27ac and survivin ChIP-seq heatmaps were generated using computeMatrix function of deepTools2 [48], version 3.5.1. Bedgraph files which contained the peak score corresponding to each ChIP-seq modification was used as the input to the computeMatrix function, and the bed file of BvCR was used as input to the computeMatrix function for the regions to be plotted. Using the scale-regions mode of the computeMatrix function, all BvCR, regardless of their width, were scaled to fit within a width of 500 bases, with a 2 kb window upstream and downstream of the BvCR. Missing peak scores were converted to zero and a 50 bp length was used for defining the score over the length of the BvCR, suggested as the default in computeMatrix function. The heatmap displays the maximum of the peak score over the length of the BvCR. The heatmaps were generated using the plotHeatmap function of deepTools2. BvCR were sorted in descending order of peak score, and the heatmap intensity was set to 50 for all the heatmaps to enable easy comparison across all histone H3 modifications. Parameters were set to default values. To examine if YM155 treatment had differential effects within and outside the BvCR, bigWigCompare function was used, which compares two bigwig files based on the number of mapped reads, where the genome is divided into several bins, and the mapped reads is counted for each bin in each of the bigwig files. Fold change was calculated for all ChIP-seq peaks of the H3 modifications or only those ChIP-seq peaks within the BvCR. If the fold change was less than 1, negative of the reciprocal of the ratio was used and interpreted as the negative fold change, as suggested by developers of the tool. The resulting bigwig file was used as the input score file for computeMatrix function and all H3 ChIP-seq peaks or the H3 ChIP-seq peaks only within BvCR were plotted using the mean of the fold change. Reference-point mode of deepTools2 was used and set to the center of the BvCR, and the fold change profile was plotted using plotHeatmap.

#### DNA motif enrichment analysis

Input bed files of survivin outside BvCR, and BvCR with and without survivin were used to retrieve FASTA sequences using the web service version of Regulatory Sequence Analysis Tools [49] and the parameters of GRCh38 as the genome organism and repeats set to masked. Using these FASTA sequences, the MEME tool of MEME suite [50] version 5.5.5 was used to identify motif sequence enrichment. Classic mode of enrichment was used, where motifs are discovered in comparison to a random model using the frequencies of the letters in the input sequence. A minimum of 10 motifs were searched, with a width between 6 and 50 bp, and enrichment was performed against the known motif database HOCOMOCO v.11 FULL [51], which contains 769 human TF binding motifs between 7 and 25 bp in width. E-value, which is an estimate of the expected number of motifs with the given log likelihood ratio (or higher), and with the same width and site count, compared to random sequences of a similar width, was used to further filter the motifs. E-value less than -100 was used, which resulted in enriched motifs for survivin and S + BvCR regions, but not for BvCR not containing survivin. The TFs found as enriched were combined and a non-redundant list was used for further analysis. FIMO tool of the MEME-Suite software, which scans sequences for motifs provided as input, was used to identify the percentage of survivin and S + BvCR regions that contained the enriched motifs. TomTom tool of MEME-Suite was used for comparison and alignment of the motifs enriched in survivin peak-containing and S + BvCR regions.

#### Peak colocalization with transcriptional regulators

To identify transcription regulators near survivin-ChIP peaks, we used the ReMap2020 database (http://remap.univ-amu.fr/) for colocalization analysis of aggregated cell- and tissue-agnostic human ChIP-seq datasets of 1034 transcriptional regulators. ReMapEnrich R-script (https://github.com/remap-cisreg/ReMapEnrich) was used for colocalization enrichment analysis.

The 4th release of ReMap [52] present the analysis of a total of 8103 quality-controlled ChIP-seq (*n* = 7895) and ChIP-exo (*n* = 208) datasets from public sources (GEO, ArrayExpress, ENCODE). The hg38 human genome assembly was used for all comparisons. Two-tailed *p* values were estimated and normalized with the Benjamini-Yekutielli test, using the maximal allowed value of shuffled genomic regions for each dataset (*n* = 15), kept on the same chromosome (shuffling genomic regions parameter byChrom = TRUE). The default fraction of minimal overlap for input and catalogue intervals was set to 10%.

#### Analysis of candidate partner TFs

To identify enrichment of the BRG1/SWI complex proteins within ReMap2022, we performed enrichment of our BvCR against the catalogue of all ReMap2022 ChIP-seq datasets. The BRG1/SWI complex proteins, based on the list provided in a recent review [12], were identified in this analysis and further filtered with a minimum number of overlaps > 5 and q-value of less than 0.05. Boxplots of q-significance, defined as the negative log10 of the q-value, were plotted. To identify the overlaps of the SWI complex with BvCR, we downloaded the entire available list of ChIP-seq datasets for the SWI complex proteins and performed overlap analysis using ChIPpeakAnno with the same parameters as mentioned previously.

#### Genomic regulatory element colocalization and overlap with TF target genes

The well-curated and robust list of experimentally confirmed candidate regulatory elements was obtained through the GeneHancer database version 5.9 by request [53]. The BED files were combined with BED files of gene bodies and 2 kb upstream promoters of hg38. Genomic locations of the regulatory elements were overlapped with genome locations of overlapping BvCR using ChIPPeakAnno package with parameters mentioned previously. The entire list of the genes connected to regulatory elements harboring BvCR was retrieved and filtered on the dual criteria of expression (base mean > 1, protein-coding) in CD4^+^T cells.

#### Construction of linear model

To investigate the relationship between survivin-dependent tag deposition of histone H3 marks and survivin-sensitive gene transcription, we constructed a linear model between the minimal, median, and maximal values of the observed tag deposition change after YM155 treatment, and the observed transcriptional change through RNA-seq. A line of best-fit, using the min–max approach, was drawn using the coordinates of these three points. Using the slope and the intercept of this line, we predicted the tag percentage for each observed fold change and calculated the difference between the predicted and observed fold changes. If this difference fell within one standard deviation of all the predicted vs observed fold changes, these genes were included in the model. For these included genes, Spearman correlation was calculated using ‘stat_cor’ function in the ‘ggpubr’ R package. Correlation modelling was performed for either all BvCR or BvCR containing survivin colocalization i.e., S + BvCR. Correlations were calculated separately for upregulated genes and downregulated genes. Radar plots were generating using the ‘ggradar’ R package.

#### Pathway enrichment analysis

Pathway enrichment analysis was performed using ShinyGO version 0.77 [54], restricting the search space to the pathway size of 100 to 1500 genes in human GO:Biological Process terms, and FDR < 0.05. The top 20 enriched terms were selected and further filtered based on the redundancy in term definition and the number of genes annotated to the enriched terms. For pathway enrichment analysis in BRG1^hi^ and BRG1^lo^ cells through the WGCNA approach, the web version of Enrichr [55] was used, restricting the search space within GO:Biological Process and Reactome2022. (reactome.org).

#### DNA Damage Response network analysis

The network map detailing subprocesses of the DNA damage response (DDR) was retrieved from a recently published affinity purification-mass spectrometry study that catalogued the protein–protein interactions of 21 central DNA damage response proteins [15]. Genes controlled by the BvCR were extracted from the original network consisting of 605 genes split into 109 hierarchical categories. The dominant H3 mark in each category was estimated by the average tag percentage of the H3 mark within the BvCR.

#### Weighted Gene Correlation Network Analysis (WGCNA)

To identify modules of genes that associated with BRG1-high and BRG1-low cells, we performed the Weighted Gene Correlation Network Analysis using the R package WGCNA [56] version 1.72–5. The matrix of normalized gene expression values of genes connected to BvCR and enriched in at least one of the pathways was used as input for WGCNA. A soft-threshold power of 10 was chosen as it was the lowest power at which the fit index reached 90%. The ‘signed’ network type was used to identify genes positively correlated (Pearson correlation) in samples having high and low BRG1 expression. A minimum module size of 30 was used with ‘mergeCutHeight’ set to 0.25. This resulted in three modules of which one module contained only 4 genes. We explored the module-gene relationships and found that this module showed correlation profiles similar to that of the module containing positively correlated genes in BRG1^lo^ samples. Hence, we merged the genes within these modules and performed pathway enrichment analysis of the resulting two modules as detailed above.

#### Compositional analysis of BRG1/SWI complex subunits

The peptides from the survivin binding custom designed microarray (*n* > 5250, PEPperCHIP, Heidelberg, Germany) comprised a scikit-learn python library to implement the machine learning process, in which the peptides were divided into equally large training and test sets [31]. Based on the functional composition of the protein, defined by the presence of atomic groups (C, CH, CH_2_, CH_3_, hydroxyl, phenyl, carboxyl, amide, sulfhydryl, etc.) rather than the sequence of amino acids, we develop a strategy for predicting fitness of a given protein/peptide to survivin in biological and a chemical context [31, 57] with the following adjustments. The multilayer perceptron classifier comprised two intermediate layer neurons, and C-Pos encoding was employed to interpret 15 amino acid-long peptides. Peptides with a C-terminal cysteine were excluded from the training data, and 90% of the original survivin peptide microarray data [58] was utilized for training purposes. The standard scaling of the training dataset for scaling the unknown datasets was applied.

The sequence data of the canonical and polybromo BRG1/SWI subunits was segmented into 15 amino acid-long peptides with 10 amino acid overlaps. Subsequently, the trained model was utilized to predict the wild-type sequence, enabling the calculation of R_bind_ (the ratio of predicted binding peptides to the total number of generated peptides). M_bind_(n) values were computed for each amino acid position by mutating the position to all other amino acids and assessing whether the machine learning model predicted survivin binding. M_bind_(n) was defined as the ratio of the total number of predicted mutant peptides (for a specific position) to the total generated mutations.

### Data analysis and visualization

Statistical analysis was performed using R-studio (version 4.3.1). Heatmaps were visualized using the R package ComplexHeatmap [59] version 2.16.0. Schematic visualizations were created using biorender.com.

## Results

### Bivalent chromatin assimilates genome deposition of survivin

**Fig. 1:**
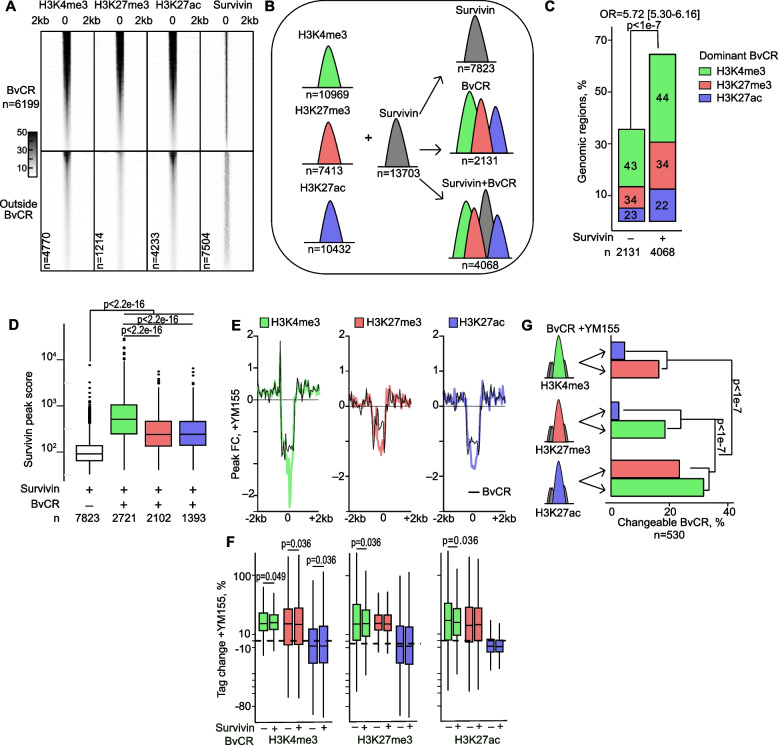
Survivin accumulates in H3K4me3-dominant bivalent chromatin regions in primary CD4^+^ cells. **A** Heatmap of ChIP-seq peaks within and outside the bivalent chromatin regions (BvCR) defined by genomic overlap between the histone H3-marks. **B** BvCR and survivin deposition. **C** Frequency difference of BvCR without and with survivin. Chi-square test *p*-value is shown. Numbers within bars indicate percentage of BvCR dominant in individual H3 marks. **D** Box plots of survivin peak scores within BvCR dominant by H3K4me3, H3K27me3 and H3K27ac marks. Kolmogorov–Smirnov test *p*-values are shown. **E** Signal plot of fold change in mean H3 peak score in 2 kb window from peak center, in naïve and YM155-treated CD4^+^ cells within (black) and outside BvCR (colored). **F** Box plot of percentage tag change in H3 modifications after YM155 treatment, within BvCR dominant in H3K4me3 (green), H3K27me3 (red), and H3K27ac (violet) within and outside survivin colocalization. Wilcoxon unpaired *p*-values are indicated. **G** Frequency of changeable BvCR that shifts in dominant H3 after YM155 treatment. Kolmogorov–Smirnov test *p*-values are shown

Annotation of chromatin bound to H3K4me3, H3K27me3, and H3K27ac, revealed a total of 6199 bivalent chromatin regions (BvCR) across the genome of CD4 + cells (Fig. 1A). The genome location of survivin peaks (*n* = 13,703, Fig. 1A) was found within 65% of BvCR (S + BvCR, 4068/6199 regions) (Fig. 1B, C). We found that, H3K4me3 mark dominated the BvCR (43%, H3K4me3-BvCR), followed by H3K27me3 (33%, H3K27me3-BvCR) and H3K27ac (24%, H3K27ac-BvCR) (Fig. 1C, Supporting Figure S2A). This frequency distribution of the dominant H3 mark was comparable for the BvCR and S + BvCR (Fig. 1C).

Investigating strength of the survivin peak binding, we found that the average survivin peak score was higher in S + BvCR, compared to survivin peaks only. Besides, survivin peaks within BvCR were significantly larger in the H3K4me3-BvCR (Fig. 1D). Similarly, score of the individual H3-marks showed the highest peak scores within H3K4me3-BvCR and a significant difference in H3-marks deposition within S + BvCR compared to BvCR not confined to survivin (Supporting Figure S2B). Notably, presence of survivin within H3K4me3-BvCR resulted in lower tag deposition of H3K4me3, H3K27me3 and H3K27ac. Presence of survivin in H3K27me3-BvCR and H3K27ac-BvCR increased deposition of H3K27me3 in those regions. Together, these results demonstrated non-randomness of survivin binding across the genome being frequently annotated to BvCR, where deposition of survivin reciprocally adjusted H3-mark deposition, appreciably in H3K4me3-BvCR.

### Binding of survivin to H3K4me3-BvCR regulates their functional status

Exploring survivin function within BvCR, we asked if survivin inhibition affected the deposition of individual histone H3 marks. To investigate this, we cultured CD4 + cells in presence of the survivin inhibitor YM155 and performed the chromatin sequencing analysis of H3K4me3, H3K27me3 and H3K27ac deposition. The adjusted average enrichment profile of BvCR showed that YM155-treated cells changed the deposition of all three H3 marks (Fig. 1E). Quantifying H3-marks deposition within BvCR of YM155-treated CD4 + cells, we observed a significant increase in deposition of all 3 modifications within H3K4me3-BvCR (Fig. 1F, the boxes are above the dotted line). This increase was more profound in the BvCR co-localized with survivin (Fig. 1F). In contrast, H3K27me3-BvCR and H3K27ac-BvCR responded to YM155 treatment by increasing deposition of H3K4me3 mark alone (Fig. 1F). Occasionally, the quantitative increase in H3 deposition caused a shift in the dominant H3 mark within BvCR (Fig. 1G). In total, such a shift occurred in 530 BvCR (8.55%) and was significantly less prevalent among the S + BvCR (325/4068 vs 205/2131, *p*-value = 0.03). We observed that the H3K4me3-BvCR or H3K27me3-BvCR shifted into each other in equal frequency, reflecting the functional bivalency of those chromatin regions (Fig. 1G). The H3K27ac-BvCR frequently lost their status and gained the dominance of H3K4me3 (32%) or H3K27me3 (23%). Therefore, the analysis of YM155-treated CD4^+^ cells showed that survivin inhibition increased the density of the lysine trimethylation on histone H3, largely increasing the proportion of H3K4me3-BvCR.

### H3K4me3-BvCR control the DNA damage response

**Fig. 2:**
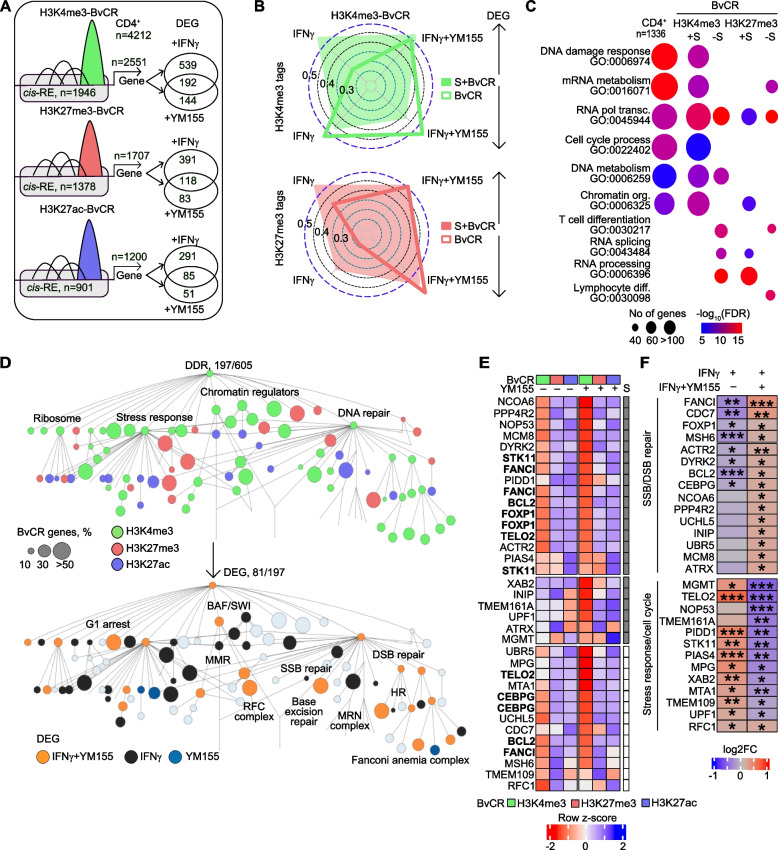
BvCR dominant in H3K4me3 together with survivin regulate transcription of DNA damage response genes. **A** Analysis strategy. BvCR within genomic regulatory elements (*cis*-RE, grey boxes) connected to genes, filtered on the protein-coding genes expressed in CD4^+^ cells, by RNA-seq. Transcription difference in CD4^+^ cells treated with IFNg or IFNγ + YM155 compared to sham cultures was calculated by DESeq2. Differentially expressed genes (DEG) were defined by a nominal *p*-value < 0.05. **B** Radar plot of Spearman’s rho correlations between H3K4me3 and H3K27me3 tag deposition change in all and survivin-positive BvCR and transcription change in CD4^+^ cells treated with IFNγ or IFNγ + YM155. Arrows indicate direction of transcription change. **C** Bubble plot of enrichment in biological processes among CD4.^+^ expressed genes connected to all and survivin-positive BvCR. Bubble size indicates protein number in the process. Color intensity shows false discovery range (FDR). **D** DNA damage response (DDR) network. Nodes are colored by dominant H3 mark in BvCR connected to genes within nodes (top map) and by transcription change after IFNγ or YM155 treatment (bottom map). Size of bubble corresponds to percentage of BvCR-connected genes within each node. DDR, DNA damage response. MMR, mismatch repair. RFC, replicator factor C. SSB, single strand break. DSB, double-strand break. HR, homologous recombination. MRN, MRE11-RAD50-NBS1. **E** Heatmap of normalized tag deposition of H3 marks, by ChIP-seq, in BvCR connected to DEG treated with IFNγ + YM155. Shaded squares indicate survivin-positive BvCR. Genes connected to multiple BvCR are marked in bold. **F** Heatmap of RNA-seq transcription difference in genes annotated to DNA repair and stress response categories. Transcription difference was calculated by DESeq2 statistics, *p*-values * < 0.05, ** < 0.01, *** < 0.001

To connect BvCR and long-distance gene regulation, we exploited the GeneHancer database [53] of experimentally confirmed connections between *cis*-RE and genes. We found that 59–65% of BvCR were located within *cis*-RE (Fig. 2A). Focusing on the transcriptome of CD4^+^ cells, we identified 4212 protein-coding genes connected to the *cis*-RE containing BvCR (Fig. 2A). Consistent with the highest frequency, the H3K4me3-BvCR had the largest number of connected genes transcribed in CD4^+^ cells (Fig. 2A).

To decipher if functional changes in the BvCR affected transcription of the connected genes, we investigated response of these genes to survivin inhibition after IFNγ stimulation. Analyzing the transcriptome in CD4^+^ cells, we found that 40% of the BvCR-connected genes were differentially expressed after IFNγ and/or YM155 treatment, i.e., were IFNγ- and/or survivin-sensitive (Fig. 2A). The changeable BvCR were less frequent among S + BvCR, while those connected to both the IFNγ-sensitive and survivin-sensitive genes were significantly predominant among S + BvCR (Supporting Figure S3A) reiterating our previous report [25] that genomic localization of survivin mediated IFNγ-dependent transcription.

To validate internal relation between the YM155-induced changes in H3 tag deposition in BvCR and the connected gene transcription, we built a linear regression model between these parameters in the IFNγ- and YM155-treated CD4^+^ cells compared to mock. Upregulation of IFNγ-sensitive and survivin-sensitive genes connected to the survivin-positive H3K4me3-BvCR had a strong direct correlation (Spearman r > 0.5) to the change in H3K4me3 and H3K27me3 tag deposition (Fig. 2B, Supporting Figure S3B), suggesting that survivin contributed to the dynamics of H3 tail deposition. Within H3K27me3-BvCR, the correlation between transcription and the survivin-dependent H3 tag deposition change was weaker (Supporting Figure S3C). These findings clearly demonstrated that 1) H3K4me3 was the epigenetic mark transducing survivin deposition in *cis-*RE into IFNγ-sensitive and survivin-sensitive regulation of transcription, and 2) transcription of survivin-sensitive genes was dependent on an interplay between H3K4me3 and H3K27me3 deposition in H3K4me3-BvCR.

To explore the cellular functions regulated by the BvCR, we searched for biological processes engaging 4212 BvCR-connected genes in CD4^+^ cells. We discovered that the DNA damage response (DDR, GO:0006974) was the principal pathway regulated by the BvCR (Fig. 2C, Supporting Table T1) followed by the nucleosome-modifying processes including cell cycle process (GO:0022402), mRNA metabolism (GO:0016071), RNA polymerase-dependent transcription (GO:0045944), DNA metabolism (GO:0006259), and chromatin organization (GO:0006325) (Fig. 2C). Consistent with a strong correlation between transcription and H3K4me3 deposition change, the genes active in these six processes were connected to the survivin-positive H3K4me3-BvCR. Such a connection was neither found in the H3K4me3-BvCR lacking survivin deposition (Fig. 2C) nor in the H3K27me3-BvCR. On the contrary, the genes connected to H3K27me3-BvCR represented immunologically relevant processes of T cell differentiation (GO:0030217), RNA splicing (GO:0043484), and RNA processing (GO:0006396) (Fig. 2C). Further, 65% of the genes connected to H3K4me3-BvCR were annotated to any of the six pathways above (Supporting Figure S4A), including a subset of genes annotated to more than two of the pathways. These results connected H3K4me3-BvCR to regulation of the DDR pathway in CD4^+^ cells.

### H3K4me3-BvCR regulate the functional DDR network

To discriminate between specific tasks within the DDR regulated by the H3K4me3-BvCR, we utilized the recently proposed DDR interaction network [15] which employed a systems biology approach to catalogue protein–protein interactions and assign them into functional DDR assemblies (Fig. 2D). Annotation of the BvCR-connected genes to the DDR network identified 197 genes which were distributed between 89% of the network nodes (Fig. 2D, top. Supporting Table T2). Notably, 63% of the nodes were dominated by H3K4me3-BvCR connected genes, including the core nodes of DNA repair, chromatin regulators, stress response and ribosome, and contained the genes of the BRG1/SWI complex including *SMARCA4, SMARCE1*, *SMARCB1*, and the Replicator Factor C complex including *RFC1, MSH2*, and *MSH6*. Gene Ontology enrichment analysis showed that more than 60% of H3K4me3-BvCR connected genes in the DDR pathway were multifunctional (Supporting Figure S4A). For example, the genes of the BRG1/SWI complex subunits were represented in > 4 pathways, which pointed at their central role in the nucleosome-modifying processes supervised by H3K4me3-BvCR. Further, we noticed that the H3K4me3-BvCR-connected genes organized the nodes of stress response, single-strand, and double-strand break repair in the DDR network, while H3K27me3-BvCR controlled the nodes of homologous recombination through the MRE11-RAD50-NBS1 complex and p53. Ribosome and stress response nodes contained only a minor fraction of the BvCR-connected genes (Fig. 2D, top, Supporting Table T2).

Among the BvCR-controlled nodes were SSB/DSB repair including specific branches of mismatch repair genes *ATAD5, RFC1, CHTF18, MSH2, MSH6*; DNA replication genes *ATAD5, RFC1, FANCI, CHTF18, MSH2, MSH6, CTPS1, MCM8*; base excision repair *MSH2*, and nucleotide excision repair *USP7, XAB2*; Fanconi anemia complex genes *FANCI, RMI2, FANCA, MCM8*; homologous recombination genes *MRE11, XRCC3, RMI2, UIMC1*; the G1 cell cycle arrest category genes *STK11, CAB39*; and multiple stress response genes.

Survivin inhibition counteracted IFNγ effects and triggered DNA damage recognition and repair (Fig. 2D, bottom). To underpin molecular mechanisms connecting the H3K4me3 deposition with the transcriptional response to IFNγ, we retrieved the IFNγ-sensitive and survivin-sensitive genes of the DDR pathway connected to H3K4me3-BvCR (Supporting Figure S4A). We found that the majority (63–68%) of the survivin-sensitive genes (*n* = 28, Fig. 2E), and the IFNγ-sensitive genes (*n* = 45, Supporting Figure S4B) were connected to the survivin-positive H3K4me3-BvCR. Furthermore, several of the survivin-sensitive genes (*FANCI*, *STK11*, *BCL2*, *FOXP1*, *CEBPG*) and the IFNγ-sensitive genes (*VRK1, RTEL1, DOT1L, MYC, BACH1, MDM4, AXIN2, XRCC3, HIPK2, HMGN1, BRD4, PYHIN1*) were connected to > 1 H3K4me3-BvCR, which multiplied survivin control.

Analyzing *cis*-RE of the survivin and IFNγ-sensitive DDR genes connected to BvCR (*n* = 28 + 45), we found that survivin inhibition caused a significant change in H3K4me3 tag deposition in the corresponding BvCR (Fig. 2E, Supporting Figure S4B, S4D). The infrequency of H3K27me3-BvCR and H3K27ac-BvCR can be appreciated from the sparsity of tag deposition in these H3 modifications (Fig. 2E, Supporting Figure S4B). The functional bivalency in transcription of DNA repair genes is reflected by the change in tag deposition of H3K4me3-BvCR and H3K27me3-BvCR in *cis*-RE connected to these genes (Supporting Figure S5). In contrast to the whole BvCR-connected transcriptome, only a minor part of changeable H3K4me3-BvCR were connected to the DDR, indicating a stability of the DDR-related H3K4me3 mark after survivin inhibition.

Analyzing the DDR network mediated through H3K4me3-BvCR, we found that numerous IFNγ-sensitive (Supporting Figure S4C) and survivin-sensitive genes (Fig. 2F) were annotated to one or more of the DDR processes. IFNγ suppressed the SSB/DSB repair and ubiquitin response genes *FANCI, MSH6, MSH2, ATAD5* and activated the genes involved in stress response and cell cycle control *CDKN1A, STK11, RMI2, TP53, PIDD1* (Fig. 2F, Supporting Figure S4C). Inhibition of survivin by YM155 counteracted these effects of IFNγ and upregulated the DNA repair genes (Fig. 2F), suggesting that a bimodal response to IFNγ was survivin dependent. Thus, interrogation of the DDR network controlled by H3K4me3-BvCR revealed that survivin acted as a critical mediator of IFNγ effects regulating the SSB/DSB repair, stress response and cell cycle control.

### Binding of survivin to BvCR is sequence-specific and assembles TF complexes

**Fig. 3:**
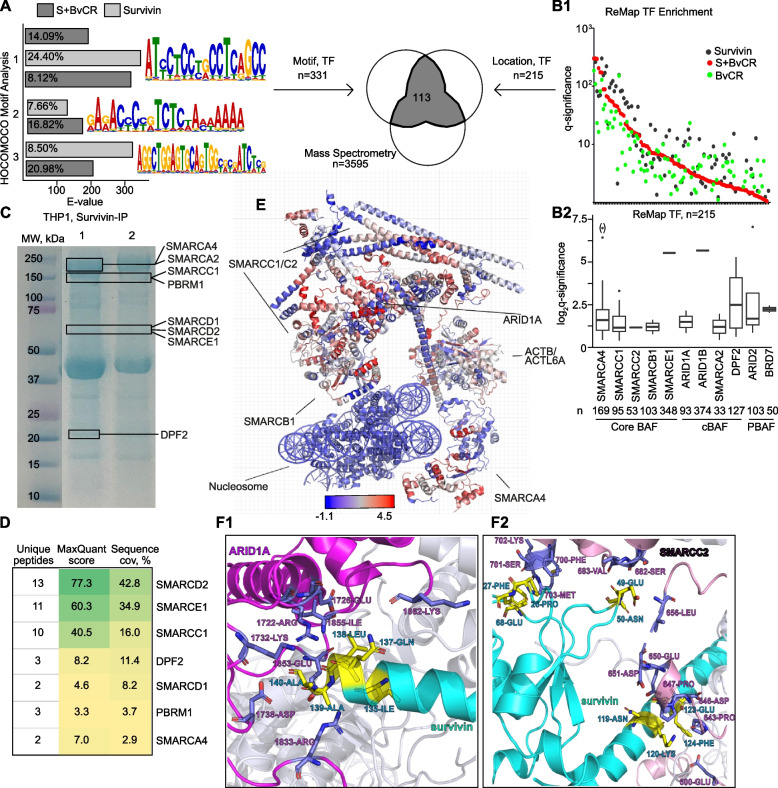
Survivin binding within H3K4me3-BvCR is sequence-specific and mobilizes BRG1/SWI complex. **A** Bar plot of motif enrichment in survivin peaks within and outside of BvCR. MEME motifs enriched within BvCR. Venn diagram of TF identified by DNA sequence motif (MEME suite) and location (ChIP-seq) and mass spectrometry (MS). Group 1 had two unique motifs with different abundances indicated by E-value. **B1** Dot plot of enrichment for human proteins/TF within BvCR, by ChIP-seq based ReMap2022 database. **B2** Box plot of enrichment for BRG1/SWI complex proteins in ReMap2022 database. Counts underneath protein names indicates the number of overlaps with BvCR. **C** Coomassie-stained electrophoresis gel depicts survivin-bound proteins precipitated from THP1 cell lysate, separated by molecular weight (MW). Lanes represent two independent experiments (1 and 2, respectively). Bands with BRG1/SWI complex proteins identified by mass spectrometry are indicated by boxes. **D** Table of BRG1/SWI subunits identified in nuclear material of THP1 cells precipitated by survivin using liquid chromatography mass spectrometry. **E** Ribbon diagram of the canonical BRG1/SWI complex (PDB ID: 6LTJ) depicts the regions binding survivin (red). Ribbon color corresponds to the normalized fluorescence intensity of survivin binding in the peptide binding array. **F** Ribbon diagram of protein–protein interaction between the human survivin-H3 tail complex (PDB ID: 3UEF) and ARID1A residues 1722-ARG, 1726-GLU, 1732-LYS, 1738-ASP, 1833-ARG, 1853-GLU, 1855-ILE, and 1862-LYS (F1) and SMARCC2 residues 600-GLU, 643-PRO, 646-ASP, 647-PRO, 650-GLU, 651-ASP, 656-LEU, 682-SER, 683-VAL, 700-PHE, 701-SER, 702-LYS, and 703-MET (F2) in the canonical BRG1/SWI complex (PDB ID: 6LTJ). Survivin, arctic blue; ARID1A, magenta; SMARCC2, rose; interaction residues, yellow

To detail survivin binding to BvCR in CD4^+^ cells, we investigated if TF assisted the reading of the histone marks. Previous studies reported that survivin controlled transcription by association with IRF1, SMAD3, and PRC2 complex [25, 31, 60]. Hence, we performed a motif enrichment analysis of the DNA sequences in the S + BvCR (*n* = 4068), survivin-negative BvCR (*n* = 2131), and the survivin peaks outside BvCR (*n* = 7823) (Fig. 3A). Analyzing 400–700 bp sequences for motif presence, we discovered that the S + BvCR and survivin peaks were frequently enriched (E-value > 100) with identical complex motifs (Fig. 3A), which were estimated as potential binding sites of 331 TF within these genome regions (Supporting Table T3). Notably, the TF motif enrichment was found solely in the survivin binding regions (S + BvCR and Survivin) and was largely absent in other BvCRs (Fig. 3A), which implied that survivin, not histone H3 modifications, accounted for the sequence specificity of the binding.

To impose on this finding, we integrated the genome location of BvCR, S + BvCR, and survivin peaks with the location occupied by human TFs identified via chromatin sequencing in a catalogue of human cells [52]. The integrated TF enrichment analysis revealed that 215 TFs had a prevalent binding within BvCR (Fig. 3B, Supporting Table T3), which was stronger in the survivin binding regions (S + BvCR and Survivin) (Fig. 3B1). The targeted search for genomic occupancy by the DDR pathway controlling BRG1/SWI complex subunits demonstrated significant overlaps with BvCR (Fig. 3B2), presenting a likelihood that survivin binding to BvCR defined genome localization of the BRG1/SWI complex. Remarkably, the core subunits of BRG1/SWI complex overlapped frequently with H3K4me3-BvCR (Supporting Figure S6A), while the subunits specific for canonical (ARID1B, DPF2, SMARCA2) and polybromo-complexes (ARID2, and BRD7) were less enriched (Fig. 3B2).

### Survivin anchors BRG1/SWI complex to BvCR

To experimentally challenge the survivin-dependent control of the DDR function, we performed protein analysis of nuclear content of THP1 cells immunoprecipitated with survivin. Survivin-IP material was separated by molecular weight using electrophoresis in two independent experiments (Supporting Figure S6B). Proteomic analysis of individual electrophoretic bands by nano LC–ESI–MS mass spectrometry revealed numerous peptides unique for the BRG1/SWI complex subunits including SMARCA2/4, SMARCC1, SMARCC2, SMARCD1, SMARCD2, SMARCE1, DPF2, PBRM1 (Fig. 3C, D) signifying a physical association of survivin with the BRG1/SWI complex. Particularly, we observed high and reproducible sequence coverage of the base module subunits SMARCC1, SMARCD1, SMARCE1, and SMARCD2 that anchored the complex to chromatin (Lane 2, Fig. 3C, D).

To detail the interaction between survivin and the BRG1/SWI complex, we applied the peptide model based on the functional group composition of each protein of the complex [31, 57]. We identified that SMARCC2, SMARCD1, SMARCA2, SMARCA4, and BRD7 exhibit comparable ratios between the predicted survivin-binding peptides to the total number of generated peptides (R_bind_-values range 0.39–0.43) and harbored a high fraction of regions favorable to survivin binding (Supporting Table T5). Introduction of mutations in the binding positions of the peptides demonstrated robustness of the predicted binding between survivin and those proteins. A value representing the ratio between the number of mutations that support binding to the total number of mutations, was mapped onto the 3D structure of canonical BRG1/SWI complex, thereby exposing stability of the survivin binding regions (Supporting Figure S6C). DPF2 and PHF10 appeared enriched in survivin-binding regions (R_bind_-values 0.46 and 0.51, respectively). Conversely, ARID1A and ARID2 exhibited low survivin-binding ratio comparable to the median of the proteome (Supporting Table T5).

Motivated by the peptide-based prediction, we performed a direct interaction experiment between survivin and BRG1/SWI complex subunits through a peptide-binding array covering the complete sequence of BRG1, ACTL6A, ARID1A, SMARCC2, SMARCD1, SMARCB1, SMARCE1, DPF2, and PHF10 proteins. The fluorescence signal generated by labeled survivin was strongest with the peptides of core subunits BRG1, SMARCC2, SMARCE1, and subunits of the canonical BRG1/SWI complex ARID1A, and DPF2 proteins (Fig. 3E, Supporting Figure S7A). The peptides of SMARCD1, SMARCB1, and PHF10 proteins had somewhat lower binding intensity although all above 30,000. The signal was observed from several peptides and formed continuous confluent regions of survivin interaction depicted in three-dimensional structure (Fig. 3E).

To visualize the most likely binding site(s) of survivin on the protein surfaces of the BRG1/SWI complex, we conducted protein–protein docking between survivin and the mammalian BRG1/SWI complexes based on the known 3D structures (cBAF, PDB ID: 6LTJ; PBAF, PDB ID: 7VDV). These docking analyses confirmed that both cBAF and PBAF use SMARCA4, SMARCC1/C2, and SMARCD1 subunits for binding with survivin. The absence of interaction between SMARCE1 and survivin in our predicted complexes may be attributed to the potential incompleteness of the EM structures of the cBAF and PBAF complexes. Additionally, cBAF-specific subunits ARID1A and DPF2 and PBAF-specific subunits PHF10, ARID2, PBRM1, and BRD7 contribute to the interactions with survivin (Supporting Figure S7A and S7B). Together, this highlighted the distinctive roles of specific BRG1/SWI subunits in chromatin accessibility regulation [61, 62]. The cBAF subunits exhibit a larger interaction area with survivin compared to the PBAF subunits (34 residues from cBAF vs. 21 residues from PBAF). Accordingly, the docking energy score for survivin binding to canonical complexes was somewhat lower compared to polybromo BRG1/SWI complexes (Supporting Figure S7A and S7B). Structural modelling of the binding interphase between the complexes and survivin demonstrated maximal binding contacts with amino acid residues of SMARCC2, SMARCD1, and ARID1A (experiment 1), SMARCB1, SMARCE1, and ARID1A (experiment 2), and ACTL6A, SMARCB1 and ARID1A (experiment 3), which supported the hypothesis that these subunits provided a probable interaction platform for survivin. Notably, the amino acid residues of SMARCC2, SMARCD1, SMARCE1 and ARID1A interacting with survivin in the docking experiments were also localized in the peptides with strongest binding fluorescent intensity to survivin in the peptide array. Based on the protein–protein docking analysis, we propose specific residues involved in the interactions between survivin and the ARID1A and SMARCC2 subunits, respectively (Fig. 3F1, F2. Supporting Figure S7A and S7B). Essentially, survivin binds the SANT-domain of SMARCC2 responsible for assembly, and stability of BRG1/SWI complexes [61, 62]. Canonical subunit ARID1A, and polybromo-specific subunit PHF10, contributed differently to the compositionally predicted and physically confirmed interactions with survivin. Interaction between ARID1A and survivin occurred within the region containing the LXXLL nuclear receptor recognition motif important for its role in gene regulation, cell biology and disease [63]. The amino acid residues of DPF2, ARID2, PBRM1, and BRD7 subunits had predicted interaction with survivin in the docking simulations, despite that their composition compatibility with survivin was low. Together, the combination of biomolecular interaction experiments through mass spectrometry, compositional analysis, peptide binding, and structural modelling, advocates in favor of survivin binding with BRG1/SWI complex through the DNA anchoring module. The differences in binding residues observed between protein docking experiments and peptide array experiments could be attributed to the structural rigidity of the BRG1/SWI complex structures which limit their ability to undergo conformational changes of the interaction regions. As a result, survivin is only able to interact with residues on the surface of these complexes.

### Inhibition of survivin and JAK-STAT signaling caused cell cycle arrest and enhanced the DNA damage recognition

**Fig. 4:**
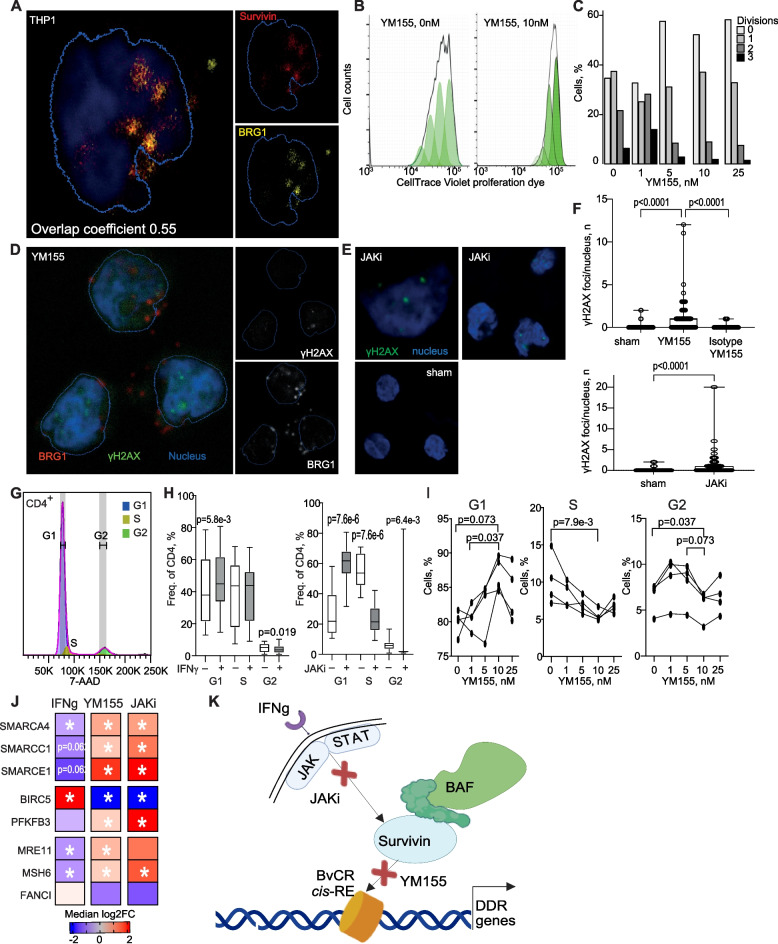
Survivin inhibition and JAKi treatment disrupts cell cycle progression and enhances DNA damage repair. **A** Colocalization of survivin (red) and BRG1 (yellow) in nucleus (blue) of THP1 cells, visualized by confocal microscopy at resolution 40X. Nuclear area is identified by Hoechst stain. Colocalization is calculated by overlap of fluorescence pixels, in ImageJ JACoB plugin. **B** Representative histogram of cell proliferation in THP1 cells treated with YM155 (0 nM and 10 nM). (right) Bar plot of THP1 cells proliferation cultured with survivin inhibitor YM155 (0–25 nM) for 72 h. Dilution of CellTrace Violet (CTV) proliferation dye was used to monitor generations of proliferating cells. **D**, **E** THP1 cells were treated with YM155 (20 nM), JAKi (50 µM), or sham (DMSO) for 24 h, fixed and stained for DNA damage using antibodies against BRG1 (red), γH2AX (green) and nuclear stain (blue). **F** γH2AX foci were counted in each nucleus of 90–200 cells per treatment using PepSlide Analyzer in ImageJ. *P*-values are obtained by Mann–Whitney U test. **G** Representative histogram of 7AAD^+^CD4^+^ cell distribution by phases of the cell cycle created by FlowJo software. Colored areas indicate G1 (blue), S (yellow) and G2 (green) phases. **H** Frequency distribution of 7AAD^+^ cells by cell cycle phases in CD4^+^ cells treated with IFNγ (50 ng/ml), and JAKi (10 µM) compared to sham (DMSO). *P*-values are obtained by Wilcoxon paired test. **I** Frequency distribution of 7AAD^+^ cells by cell cycle phases in CD4^+^ cells treated with YM155. *P*-values are obtained by Friedman test. **J** Heatmap of qPCR gene expression change by median log2FC in CD4^+^ cells treated with IFNγ (50 ng/ml), YM155 (10 nM) or JAKi (10 mM) for 48 h, compared to control (DMSO), measured by qPCR. Asterisk indicates *p*-values calculated by Wilcoxon paired test. * < 0.05. **K** A model of intervention in IFNγ signaling, IFN, survivin-BRG1 complex and BvCR dependent transcription of DNA damage response (DDR) genes by treatment with JAK-inhibitor (JAKi) and survivin inhibitor YM155. *cis*-RE, regulatory element

To examine colocalization of survivin and the BRG1/SWI complex, we performed immuno-histochemistry targeting BRG1 and survivin. We used BRG1 as a representative for the whole complex as BRG1 is the catalytic subunit and therefore a fixed component of all type of BRG1/SWI complexes. The convergence of survivin and BRG1 in nuclear and peri-nuclear area was visualized in THP1 cells known for abundant survivin expression. The analysis was performed on a pixel-by-pixel basis and presented 33–88% overlap in fluorescence produced by survivin and BRG1 (Fig. 4A, Supporting Figure S8).

Based on the acquired results, we hypothesized that the DDR network in CD4^+^ cells was controlled by S + BvCR located within *cis*-RE of the human genome. To aid this function, survivin anchored the BRG1/SWI complex to the BvCR via transduction of the activating effects of IFNγ through JAK-STAT signaling.

To gather experimental verification of this model, we investigated the role of survivin in cell proliferation and cell cycle progress. For this, we cultured THP1 cells in presence of survivin inhibitor YM155 for 72 h. The CellTrace Violet dye was added to the culture medium to monitor new generations of proliferating cells by dye dilution. We found that survivin inhibition resulted in a decreased frequency of new generations of THP1 cells clearly seen in YM155 concentrations above 5 nM, while the part of maternal undivided cells increased (Fig. 4B, C). Additionally, survivin inhibition by YM155 promoted the accumulation of phosphorylated histone γH2AX^+^ foci which recognized the double-strand breaks in THP1 cells (Fig. 4D, F). A similar accumulation of γH2AX foci was observed after JAK/STAT inhibition (Fig. 4 E, F).

Next, we studied the cell cycle progress in CD4^+^ cells using the DNA binding fluorescent dye in flow cytometry (Fig. 4G). We observed that the progression of cell cycle was opposed by inhibition of survivin using YM155 causing a significant and dose dependent retention of cells in G1 phase and depleting cells in S and G2 phases, consistent with cell cycle arrest (Fig. 4I). Similar alterations in cell cycle progress were induced by inhibition of JAK/STAT signal downstream of IFNγ receptor (Fig. 4H). Therefore, both survivin and JAK/STAT inhibition disrupted proper cell cycle progression in CD4^+^ cells.

The IFNγ stimulation activated the functional link between survivin and energy supply in CD4^+^ cells by upregulating *BIRC5* and repressing *PFKFB3* (Fig. 4J) [25], the gene important for efficient homologous recombination [64]. Notably, the IFNγ-treated cells reduced mRNA levels of *SMARCA4*, *SMARCC1*, *SMARCE1*, which was associated with low transcription of the central DNA repair genes *MRE11*, *MSH6*, and *FANCI* connected to the H3K4me3-BvCR in CD4^+^ cells (Fig. 4J). This effect of IFNγ on DNA repair genes was abrogated by inhibition of survivin or JAK-STAT signaling (Fig. 4J).

Together, the in vitro studies validated the proposed hypothesis in which survivin exploited bivalency in aiding IFNγ signaling and BRG1/SWI complex in the DDR activity. Inhibition of IFNγ signaling and survivin was associated with cell cycle arrest and accumulation of damaged DNA (Fig. 4K). This triggered the subsequent activation of DNA repair genes, including transcription of the BRG1/SWI complex subunits.

### BRG1 expression defined a specific phenotype of CD4^+^ cells in patients with rheumatoid arthritis

**Fig. 5:**
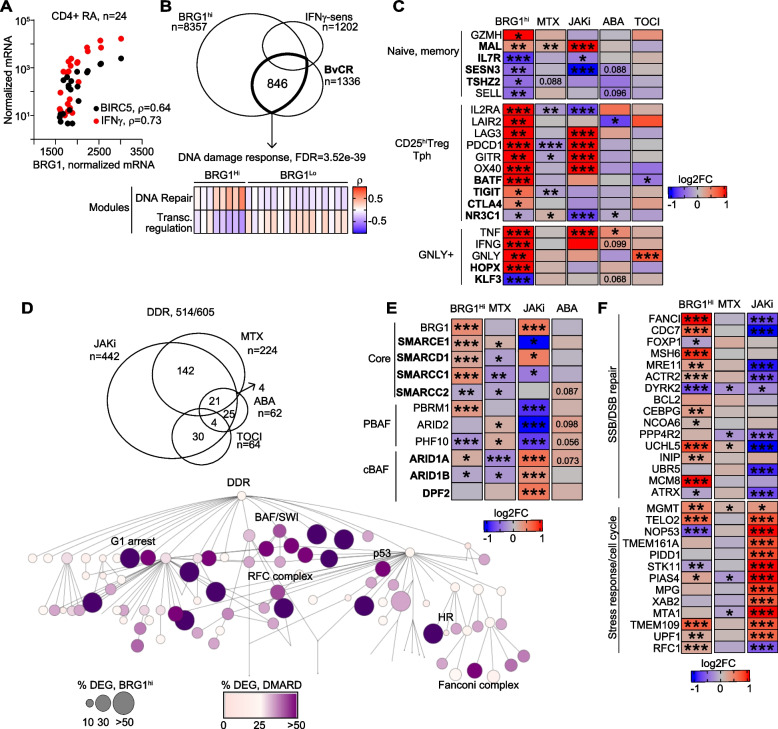
Immunomodulating treatment affects DNA damage response in CD4^+^ cells of patients with rheumatoid arthritis. **A** Dot correlation plot of normalized mean expression of *BRG1, BIRC5* and *IFNG* genes in CD4^+^ cells of patients with rheumatoid arthritis. Spearman’s rho values are indicated. **B** Venn diagram of differentially expressed genes (DEG) in BRG1^hi^CD4^+^ cells connected to BvCR and in IFNγ-treated CD4^+^ cells. Heatmap of Spearman’s rho correlation values of genes connected to BvCR in BRG1^hi^ and BRG1^lo^ cells identified by weighted correlation network analysis (WGCNA). **C** Heatmap of expression difference in T cell specific markers identified by RNA-seq in BRG1^hi^CD4^+^ cells and in CD4^+^ cells before and after treatment with abatacept (ABAT, *n* = 14), tocilizumab (TOCI, *n* = 6) and methotrexate (MTX, *n* = 28) and in CD4^+^ cells of JAKi-treated (*n* = 23) and untreated (*n* = 9) RA patients. Expression difference in CD4^+^ cells of treated and untreated patients was calculated by DESeq2. Nominal *p*-values are indicated. * < 0.05, ** < 0.01, *** < 0.001. **D** Venn diagram of DEG changed with treatment in the DNA damage response (DDR) network. DDR network map of DEG changed with treatment. Node size indicates the percentage of BRG1^hi^ DEG. Node color indicates the percentage of DEG in the node. **E** Heatmap of expression difference in BRG1/SWI complex proteins in BRG1^hi^CD4^+^ cells and in CD4^+^ cells after treatment, by RNA-seq. Expression difference was calculated by DESeq2. Nominal *p*-values are indicated. * < 0.05, ** < 0.01, *** < 0.001. **F** Heatmap of expression difference in DDR network genes in BRG1^hi^CD4^+^ cells and in CD4^+^ cells after treatment

To investigate the impact of the BRG1/SWI complex in the survivin-dependent DDR control in autoimmune cells, we used transcriptome datasets of CD4^+^ cells of 24 RA patients (Supporting Fig. 1A). Guided by BRG1*/SMARCA4* transcription, we found that both survivin and IFNγ were highly co-expressed in the *BRG1*^*hi*^CD4^+^ cells (Fig. 5A). Mapping of the differentially expressed genes to the DDR network revealed that the nodes of DNA repair, replication, and G1 arrest were upregulated in BRG1^hi^ cells (Supporting Figure S9), pointing at unbalanced DDR control in these cells.

Next, we investigated if BvCR-connected genes abnormally regulated in BRG1^hi^ cells were associated with the pathogenic phenotype in RA CD4^+^ cells. A total of 63% (846/1336) of the genes involved in at least one of the main pathways controlled by BvCR were differentially expressed in BRG1^hi^CD4^+^ cells, including the DDR pathway (Fig. 5B). To identify the functional modules of the genes co-expressed with BRG1, we applied the weighted gene correlation network analysis (WGCNA), asking if the hub genes that showed high co-expression in the BRG1^hi^ and BRG1^lo^ cells were functional in the BvCR-controlled biological processes. The WGCNA approach identified two modules, where the hub genes were positively associated with BRG1^hi^ cells (*n* = 498) and with BRG1^lo^ cells (*n* = 348) (Fig. 5B, Supporting Table T4). Several core subunits of the BRG1/SWI complex including BRG1/*SMARCA4, SMARCC1, SMARCD1, SMARCE1* and canonical subunit *ARID1A* were accumulated in the module of BRG1^hi^ cells (Fig. 5E). Additionally, this module showed the enrichment for the DNA repair pathway (GO:0006281, FDR = 2.8e-34) and cell cycle pathway (R-HSA-1640170, FDR = 1.3e-36) (Fig. 5B, F). Common cell cycle genes *TP53* and *CDKN1A* controlled by BvCR were also highly upregulated in the *BRG1*^*hi*^ cells while the *ATM* gene was repressed. The module of BRG1^hi^ cells included the IFNγ-sensitive genes of *PIAS4*, *MSH6*, *FANCI*, *MRE11, TELO2*, and *SMC3* (Fig. 5F) that changed H3K4me3 tag deposition after survivin inhibition (Fig. 2F, Supporting Figure S5). Several regulators of transcription including *FOXP1, CBX4, PBX1, LEF1* were repressed and present in the module of BRG1^lo^ cells (FDR = 1.35e-70).

To translate the profile of BRG1^hi^CD4^+^ cells into joint pathology in rheumatoid arthritis, we utilized characteristics of the inflammatory cells identified in RA synovia with a single cell resolution [65]. With focus on CD4^+^ cells, we investigated if the key markers of the synovial clusters were differentially expressed in the BRG1^hi^CD4^+^ cells and connected to BvCR. We found that transcriptome of *BRG1*^*hi*^CD4^+^ cells was enriched in the synovial cytotoxic *GNLY*^*hi*^*HOPX*^*hi*^ cells expressing *IFNG, TNF* and *GZMA*, and the peripheral T-helper cells abundant in the immune check-point receptors PD1*/PDCD1*, *CTLA4*, and *LAG3* (Fig. 5C) and TNF-superfamily receptors OX40/*TNFRSF4*, GITR/*TNFRSF18*, *LAIR2*, known for the ability to infiltrate inflamed tissue and to drive autoimmunity in RA [66–68]. In contrast, the naïve and memory T cell subsets recognized by *IL7R, SESN3, KLF3, NR3C1* and *TSHZ2* genes connected to the BvCR, were subdued in BRG1^hi^CD4^+^ cells. Notably, most of the synovial subset markers were both controlled by BvCR and sensitive to IFNγ (Fig. 5C). Thus, the deregulated control of the DDR pathway in BRG1^hi^CD4^+^ cells was a prominent feature of the pathogenic peripheral T-helper cells abundant in RA synovia contributing to their pathology.

### Immunomodulating treatment changed the BvCR-dependent profile of the pathogenic BRG1^hi^CD4^+^ cells

Asking how immunomodulating treatment influenced the DDR pathway in the BRG1^hi^CD4^+^ cells, we investigated the paired transcriptome data of CD4^+^ cells obtained before and after treatment with CTLA4-fusion protein abatacept [69] (*n* = 14), IL6 receptor blocking antibody tocilizumab [70] (*n* = 6), and methotrexate [71] (*n* = 28). The effect of JAK-inhibitors (JAKi) on CD4^+^ cells was studied using cross-sectional transcriptome of JAKi-treated (*n* = 24) and untreated RA patients (*n* = 9) (Supporting Figure S1A).

A total of 514 of 605 genes annotated to the DDR network were responsive to the immunomodulating treatment (Fig. 5D). The predominant part of the genes (73%) was changed in the JAKi-treated patients, methotrexate accounted for 37% of the DEG followed by abatacept and tocilizumab in equal proportions. Interestingly, > 50% of the DEG in the BRG1^hi^CD4^+^ cells that were functional in the DDR network processes of G1 arrest, p53 pathway, DNA replication by RFC complex, and Fanconi complex were responsive to at least one of the treatments (Fig. 5D). The genes of SSB/DSB repair and stress response, in which we had earlier detailed the effect of survivin and H3K4me3-BvCR (Fig. 2G, Fig. 4H), revealed the opposing effects of methotrexate and JAKi (Fig. 5F). Methotrexate changed the expression of SSB/DSB repair genes *DYRK2*, *PPP4R2* and *UCHL5* and the stress response genes *MGMT*, *MTA1*, *PIAS4* (Fig. 5F), while JAKi treatment upregulated all stress response genes and repressed the DNA repair genes (Fig. 5F). The JAKi effect was pronounced in the genes of *FANCI, CDC7, MCM8, MGMT,* and *MRE11* which reversed the expression observed in the BRG1^hi^CD4^+^ cells. This replicated effect of JAKi on the BvCR-controlled DDR observed in cultured CD4^+^ cells (Fig. 4).

Since different BRG1/SWI complexes function in specific cellular processes [62, 72], we analyzed the expression of canonical and polybromo-specific subunits after the immunomodulating treatment. Intriguingly, methotrexate and JAKi treatment had a reciprocal effect on transcription of polybromo-specific subunits *PBRM1*, *ARID2,* and *PHF10*, and canonical subunits DPF2, *ARID1A,* and *ARID1B* (Fig. 5E). Abatacept tended to upregulate both *PHF10* and *ARID1A*, while tocilizumab had no effect on either of the BRG1/SWI complex genes. The predicted survivin interactors *SMARCC1* and *SMARCD1* anchoring the BRG1/SWI complex to H3K4me3-BvCR (Fig. 3), were downregulated by both methotrexate and JAKi, which could impair BRG1/SWI complex function in recognition of DNA damage [12, 72] leading to accumulation of γH2AX on chromatin as demonstrated (Fig. 4E, F).

Taken together, immunomodulating treatment altered composition of the BRG1/SWI complex decreasing affinity of the complex to BvCR, which loosened the transcriptional control of the BvCR-connected genes active in the DDR pathway.

## Discussion

In this study, we uncover the function of BvCR in orchestrating DNA damage control and ensuring genome fidelity in adaptive immunity. Survivin appears as an essential facilitator in this process by ensuring an appropriate balance between the H3K4me3 and H3K27me3 deposition and a sequence-specific mobilization of BRG1/SWI complex to the BvCR. Through the peptide binding array, compositional analysis, and structural modelling of the docking, we predict the anchoring interaction between survivin and the BRG1/SWI complex subunits and propose their concerted action in mediating IFNγ effects on DNA damage response, further confirming the relevance of this action in autoimmune CD4^+^ cells of patients with rheumatoid arthritis. By transcriptome profiling of the BRG1^hi^CD4^+^ cells, we demonstrated their pathogenic potential linked to aberrant DNA damage response.

Our study revealed the importance of BvCR in finetuning transcriptional characteristics of distal *cis*-RE of CD4^+^ cells, adding a new function to the known role of BvCR in resolving T cell lineage choice [5, 6, 8]. Refined approach of sequential chromatin immunoprecipitation has shown that functionally antagonistic histone modifications are present at the same location [5], giving credence to our findings. We propose both abundance, and functional significance, of these modifications at specific regions of the CD4^+^ T cell genome, albeit in bulk cell population. Our data explains how survivin reads chromatin bivalency. Survivin preferably navigated to H3K4me3-BvCR and bound in a sequence-specific manner, which then opened up a platform for the BRG1/SWI complex recruitment and cooperation with TFs that read H3 marks in the BvCR [31]. Through the occupancy of BvCR, survivin balanced the quantitative level of H3K4me3 and H3K27me3 deposition in *cis-*RE. Feasibility of this hypothesis is evidenced by the high precision chromatin immunoprecipitation which ascribed H3K4me3-BvCR with regulation of genes intricate to the immune system [5]. In addition to transcription, the chromatin bound BRG1/SWI complex assists in its 3D organization and nucleosome activity by eviction and replacement of histone variants [10, 12]. Supporting this multifunctionality, we revealed a significant change of histone H3 marks after survivin inhibition and transcriptional alteration of the canonical and polybromo BRG1/SWI complexes. This phenomenon could potentially be explained by the disruption of molecular interactions between survivin and the subunits SMARCC2, and ARID1A of the BRG1/SWI complex, crucial for nucleosome binding and complex stability on chromatin. This mechanistic model is closely related to a recent functional study deducing the role of epigenetic modifications for affinity of BRG1/SWI complex to chromatin [73]. Pertinent to this, our previous report demonstrated that survivin inhibited the PRC2 repressive complex that obstructs transcription [31]. Both, in case of PRC2 and in case of BRG1/SWI, survivin binding was annotated to the SANT-domain of EZH2 and SMARCC2 proteins, respectively. The chromatin modifying complexes of BRG1/SWI and PRC2 display antagonistic purposes and are mutually exclusive at the same locus [74, 75]. Thus, survivin reads and co-opts multiple functions of the bivalent genome template by adopting mutually exclusive modes of interaction with BRG1/SWI and PRC2 complexes.

This study uncovers an unappreciated role of BRG1/SWI complex function in autoimmunity. Aberrant genome organization activates IFNγ-signaling through an increased DNA damage response, identified as one of the emerging functions of the BRG1/SWI complex [12, 72, 76]. Our study shows that survivin is an intermediary in this connection and propagates IFNγ-mediated effects in CD4^+^T cells through accelerating cell cycle progression. Survivin inhibition and inhibition of JAK/STAT signaling counteracts the IFNγ-induced effect through the cell cycle arrest and upregulation of DNA repair genes. Together with the shown association of survivin with the BRG1/SWI complex, our data support the view that survivin adjusts BRG1/SWI complex composition and location in the efficiency of DNA repair activity. These findings build on our previous study which has shown that survivin transcriptionally controls *PFKFB3* [25], the glycolytic enzyme which plays a crucial role in promoting DNA repair [64] and encouraging metabolic adaptation in IFNγ-producing CD4^+^ cells. Here, we exposed DNA damage signaling as one of the IFNγ-dependent processes in CD4^+^ cells coordinated by survivin and the BRG1/SWI complex, underscoring the obvious connection between genome organization and immune signaling pathways.

Activation of the DDR pathway is a crucial characteristic of autoimmune CD4^+^ cells [77, 78]. We found that BRG1^hi^CD4^+^ cells of RA patients combined the high survivin levels with an upregulated DNA damage profile. The BRG1^hi^CD4^+^ cells were highly enriched in the immune checkpoint receptors characteristic for the peripheral T-helper CD4^+^ cells identified in the RA synovial tissue [65]. This similarity outlines the pathogenic potential of the BRG1^hi^CD4^+^ cells in blood capable of homing to the joint to propagate arthritis. Methotrexate and JAKi treatment modulated the DNA damage signaling, potentially by eliciting a shift in composition of the BRG1/SWI complex to enact DNA damage response and downregulating the anchoring subunits *SMARCE1, SMARCD1, SMARCC2,* where survivin acted as an intermediary mobilizing BRG1/SWI complex to BvCR. Together, these processes modulate the DNA damage signaling activity in autoimmune BRG1^hi^CD4^+^ cells.

## Conclusions

Our study presents a hitherto undefined connection between BvCR, BRG1/SWI complex and survivin in CD4^+^T cells to assure the promotion of DNA damage response ubiquitous in autoimmunity. DNA repair and stress response were activated in autoimmune CD4^+^ cells of RA patients, which combined the high survivin and BRG1 levels. Intervention targeting the BRG1/SWI complex mobilization to the bivalent chromatin offers a new platform for drug development targeting autoimmunity.

## Supplementary Information

Additional file 1: Supporting Figure S1. S1A. The table of clinical characteristics of healthy controls and patient material used in this study. S1B. The table of primers used for qPCR analysis in this study. S2A. Box plots of histone H3 tag deposition within the bivalent chromatin regions (BvCR) dominant by H3K4me3, H3K27me3 and H3K27ac. S2B. Box plots of histone peak scores within BvCR dominant by H3K4me3, H3K27me3 and H3K27ac. Kolmogorov–Smirnov test *p*-values are shown. S2C. Box plot of percentage tag change for histone peaks within H3K27me3- and H3K27ac-BvCR, after YM155 treatment. Mann–Whitney test *p*-values are indicated. Supporting Figure S3. S3A. Forest plot of probability of BvCR to be changeable (Ch) in YM155-treated CD4 cells and genes connected to BvCR to be differentially expressed (DEG) in CD4 + cells treated with IFNγ or IFNγ + YM155. S3B. Scatter plot of correlation between change in deposition of H3K4me3 and H3K27me3 tags and change in transcription of DEG after treatment with IFNγ or IFNγ + YM155 in survivin-positive H3K4me3-BvCR. Spearman ρ are indicated. S3C. Radar plot of Spearman’s ρ correlations between tag change in H3K4me3 and H3K27me3 deposition in H3K27me3-BvCR and transcription change of DEG in CD4+ cells treated with IFNγ or IFNγ + YM155. Arrows indicate direction of transcription change. Supporting Figure S4. S4A. Bar plot of frequency of genes connected to BvCR in the enriched pathways. To the right is the Venn diagram of IFNγ- and survivin-sensitive genes connected to H3K4me3-BvCR and annotated to the DNA damage response pathway (GO:0006974). S4B. Heatmap of normalized tag deposition in BvCR connected to DEG treated with IFNγ + YM155. Filled squares indicate colocalization of survivin (S) in the BvCR. Genes connected to multiple BvCR are marked in bold. S4C. Heatmap of transcription change of DEG annotated to DNA Damage Response (DDR) pathway. Asterisks indicate RNAseq nominal *p*-values—* < 0.05, ** < 0.01, *** < 0.001. S4D. Box plot of quantified tag deposition in survivin-sensitive and IFNγ-sensitive genes annotated to DNA damage response and connected to H3K4me3-BvCR. Mann–Whitney *p*-values are indicated. Supporting Figure S5. Genomic maps of the DNA repair gene loci MSH6, FANCI, SMC3, PIAS4, and MRE11. Filled black and red boxes indicate cis-RE connected to the gene, as determined by GeneHancer. Distance to TSSs is shown. Black filled peaks underneath cis-RE indicate the positions of survivin-ChIP and histone H3-ChIP peaks. Colored peaks indicate the change in tag deposition for H3K4me3 (green) and H3K27me3 (red) after YM155 treatment, scaled to enable direct comparison between the two modifications. Supporting Figure S6. S6A. Frequency of overlapping BvCR with cBAF and PBAF complex subunits retrieved from ReMap2022 database. Fisher test *p*-values are indicated. S6B. Coomassie-stained electrophoresis gel depicts replicate nuclear extracts of input (lanes 2 and 3), survivin-IP (lanes 7 and 10) and non-specific IgG IP (lanes 15 and 16). The red-marked bands were excised for interrogation using LC–MS. Molecular weight ladder (MW) is shown on the left side and in lane 14. Experiment 1 is presented in lanes 2, 7 and 15; experiment 2 is presented in lanes 3, 10 and 16. Red numbers indicate the bands analyzed by mass spectrometry. S6C. Distribution of survivin binding probability across the protein sequence of SMARCC2, SMARCD1, and SMARCE1. Mbind(n) value indicate the fraction of mutations compatible with a survivin binding to the residue, defined by the functional composition of atomic group. “1” indicates a region predicted to bind survivin even if the position is mutated to any other amino acid. “0” indicates no mutation can convert the site to survivin binding region. UniProt IDs of the proteins are indicated in brackets. Supporting Figure S7. S7A. Interaction between survivin and the conventional BRG1/SWI complex (PDB ID: 6LTJ) predicted by docking modelling in three independent experiments and in the peptide-binding array. Peptide residues involved in the interaction with survivin are marked bold. S7B. Interaction between survivin and the polybromo BRG1/SWI complex (PDB ID: 7VDV) predicted by docking modelling in three independent experiments and in the peptide-binding array. Peptide residues involved in the interaction with survivin are marked bold. Supporting Figure S8. Gallery of immunohistochemical images depicting colocalization of survivin (red) and BRG1 (yellow) in nucleus (blue) of THP1 cells, visualized by confocal microscopy at resolution 40X. Nuclear area is identified by Hoechst stain. Overlap coefficient was calculated by colocalization of fluorescence pixels using ImageJ JACoP plugin. Supporting Figure S9. DNA Damage Response (DDR) network map of upregulated (red) and downregulated (blue) differentially expressed genes in BRG1hi cells in patients with rheumatoid arthritis. Nodes are colored by fold expression difference (log2FC) between BRG1hi and BRG1lo CD4+ cells of genes within nodes. Size of bubble corresponds to percentage of BRG1hi genes within each node.

Additional file 2: Supporting Table T1. Pathway enrichment (GO: BP).

Additional file 3: Supporting Table T2. DDR network analysis of the genes connected to BvCR.

Additional file 4: Supporting Table T3. Overlap of enriched TF identified through Mass Spectrometry, ReMap database, and HOCOMOCO-based enrichment.

Additional file 5: Supporting Table T4. Modules after WGCNA analysis of DEG in BRG1hi and BRG1lo cells.

Additional file 6: Supporting Table T5. Rbind values of ARID1A, ARID2, SMARCC2, SMARCD1, and SMARCE1.

## Data Availability

The datasets supporting the conclusions of this article are available in the following NCBI GEO repository. RNA- and ChIP-seq data (raw data and processed files) of CD4 + T cells that support the findings of this study have been deposited in NCBI GEO with the following identifiers – Survivin ChIP-seq: GSE190354; H3K27me3 ChIP-seq ± YM155 treatment: GSE216818; H3K27ac ChIP-seq ± YM155 treatment, GSE216817; H3K4me3 ChIP-seq ± YM155 treatment, GSE216820. RNA-seq: 1) CD4^+^T cells RA patients, GSE190349; 2) human CD4^+^T cells treated with IFN, IFN + YM155, and control, GSE190351; 3) CD4^+^T cells of RA patients treated with JAK-inhibitors and untreated, GSE201669. The mass spectrometry proteomics data of survivin affinity immunoprecipitated material have been deposited to the ProteomeXchange Consortium via the PRIDE partner repository [79] with the dataset identifier PXD049683. The peptide microarray binding data are available upon reasonable request. Other CD4^+^T cell RNA-seq datasets: CTLA4-fusion protein abatacept treatment GSE121827, IL6 receptor blocking antibody tocilizumab treatment GSE113156, and methotrexate treatment GSE176440. CD4^+^T cell clusters in RA synovial tissue by CITE-seq, https://doi.org/10.1038/s41586-023-06708-y All other relevant data supporting the key findings of this study are available within the article and its Supporting Information files.

## Abbreviations

BvCR: Bivalent Chromatin Regions
RA: Rheumatoid arthritis
PRC2: Polycomb Repressive Complex 2
DDR: DNA Damage Response
WGCNA: Weighted Gene Correlation Network Analysis
