# Supplementary material for: Bivalent chromatin accommodates survivin and BRG1/SWI complex to activate DNA damage response in CD4+ cells
Source: Cell Commun Signal. 2024 Sep 11;22:440. doi: 10.1186/s12964-024-01814-4 (PMC11389452; doi:10.1186/s12964-024-01814-4)

Supporting Figures

S1A. The table of clinical characteristics of healthy controls and patient material used in this study.

	Healthy Controls	GSE190349	GSE201669	GSE176440	GSE121827	GSE113156
n	40	24	33	28	14	6
Treatment	N/A	N/A	JAKi	Methotrexate	Abatacept	Tocilizumab
Mean age, years (range)	51.6 (26-77)	53.8 (25-73)	54.3 (23-73)	59.5 (47.5-67)	37.7 (24-58)	65 (52-67)
Female gender, %	97.5	100	100	71.4	78.6	66.7
Disease duration, median (IQR), months	N/A	66 (20-134)	126 (72-228)	84 (36-288)	N/A	67 (14-293)
ESR, median (IQR), mm/h	N/A	19.5 (8.7-25.2)	11 (5-20)	31 (14-46)	N/A	41 (13-62)
ACPA-positive, n (%)	N/A	12 (50%)	18 (54%)	26 (92.9)	N/A	4 (66.7)
RF-positive, n (%)	N/A	12 (50%)	19 (57%)	25 (89.2)	N/A	4 (66.7)

S1B. The table of primers used for qPCR analysis in this study.

	Forward	Resverse
BIRC5	GACCACCGCATCTCTACATTC	TGCTTTTTATGTTCCTCTATGGG
FANCI	CCTCCAAGGGAAGCAGAAAG	TCTCCTTGCTGTCCTACCTT
MRE11	ACAAGGAGGAGAAAGATGCCA	TCATAGCCTCACGGACTTCAT
MSH6	AGCCCTCAGAGCCAGAAGA	AATTCCACATCAGAGCCACC
PFKFB3	CCTACAACTTCTTCCGCCCC	CCGCAATTTGTCCCCCTTCT
SMARCA4	ACCAGAAGCAGAGCCGCAT	TCAATGGTCGCTTTGGTTCG
SMARCC1	AGCTGTTTATCGACGGAAGGA	CTGAAGAAGCTGCACCACC
SMARCE1	ACCATCTTATGCCCCACCTC	TCCCAGCCTGTAGTTGTTGT

28 aug. 2024

S2A. Box plots of histone H3 tag deposition within the bivalent chromatin regions (BvCR) dominant by H3K4me3, H3K27me3 and H3K27ac

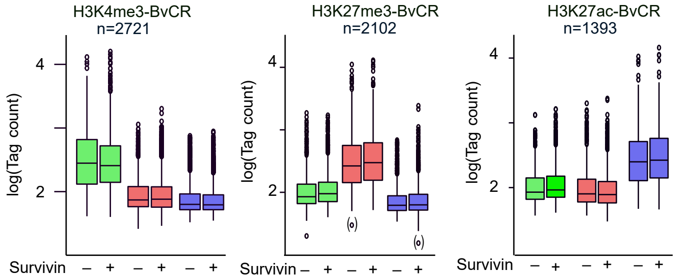

S2B. Box plots of histone peak scores within BvCR dominant by H3K4me3, H3K27me3 and H3K27ac. Kolmogorov-Smirnov test p-values are shown.

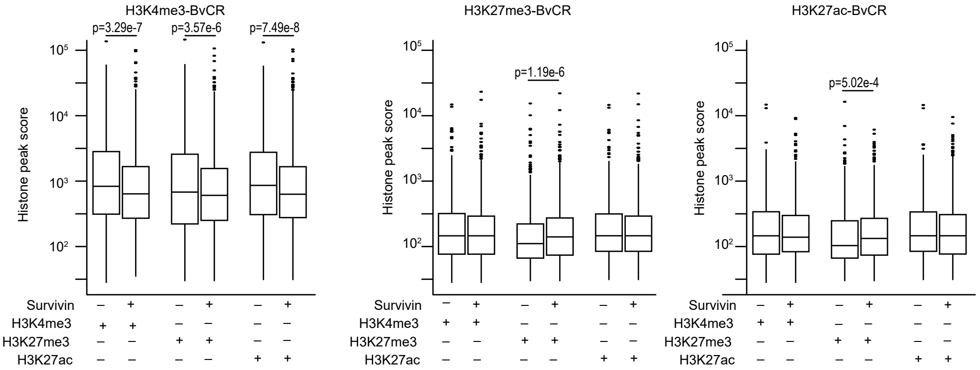

S3A. Forest plot of probability of BvCR to be changeable (Ch) in YM155-treated CD4 cells and genes connected to BvCR to be differentially expressed (DEG) in CD4+ cells treated with IFNγ or IFNγ+YM155.

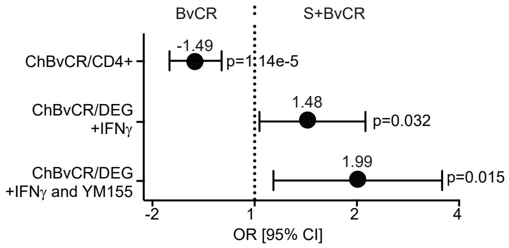

S3B. Scatter plot of correlation between change in deposition of H3K4me3 and H3K27me3 tags and change in transcription of DEG after treatment with IFNγ or IFNγ+YM155 in survivin-positive H3K4me3-BvCR. Spearman ρ are indicated.

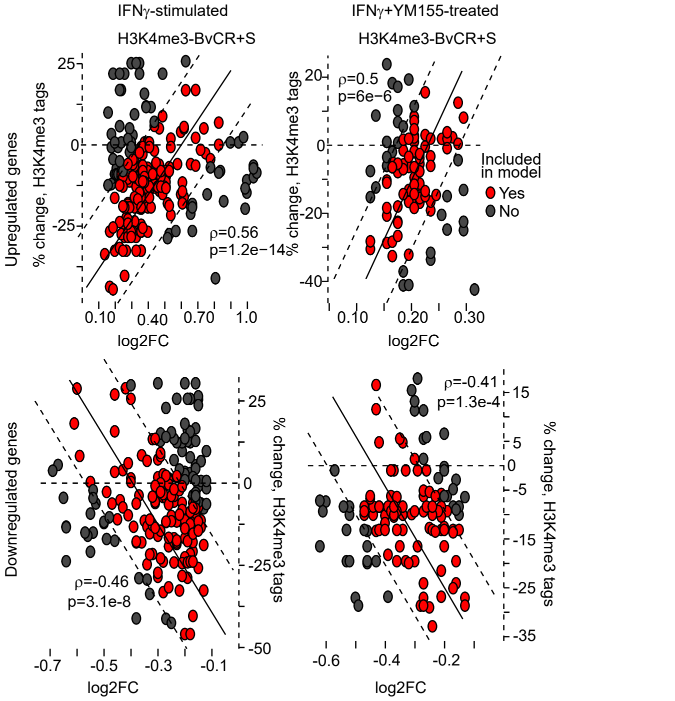

S3C. Radar plot of Spearman's ρ correlations between tag change in H3K4me3 and H3K27me3 deposition in H3K27me3-BvCR and transcription change of DEG in CD4⁺ cells treated with IFNγ or IFNγ+YM155. Arrows indicate direction of transcription change

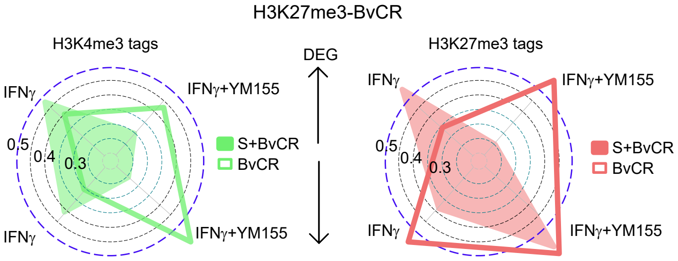

S4A. Bar plot of frequency of genes connected to BvCR in the enriched pathways. To the right is the Venn diagram of IFNγ- and survivin-sensitive genes connected to H3K4me3-BvCR and annotated to the DNA damage response pathway (GO:0006974)

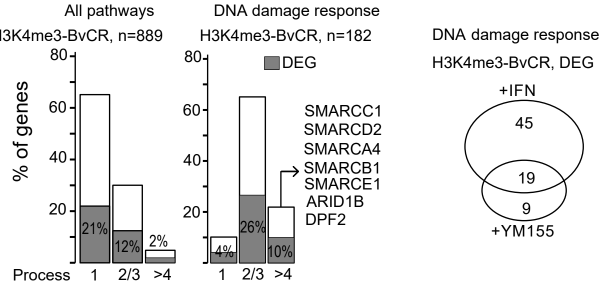

S4B. Heatmap of normalized tag deposition in BvCR connected to DEG treated with IFNγ+YM155. Filled squares indicate colocalization of survivin (S) in the BvCR. Genes connected to multiple BvCR are marked in bold

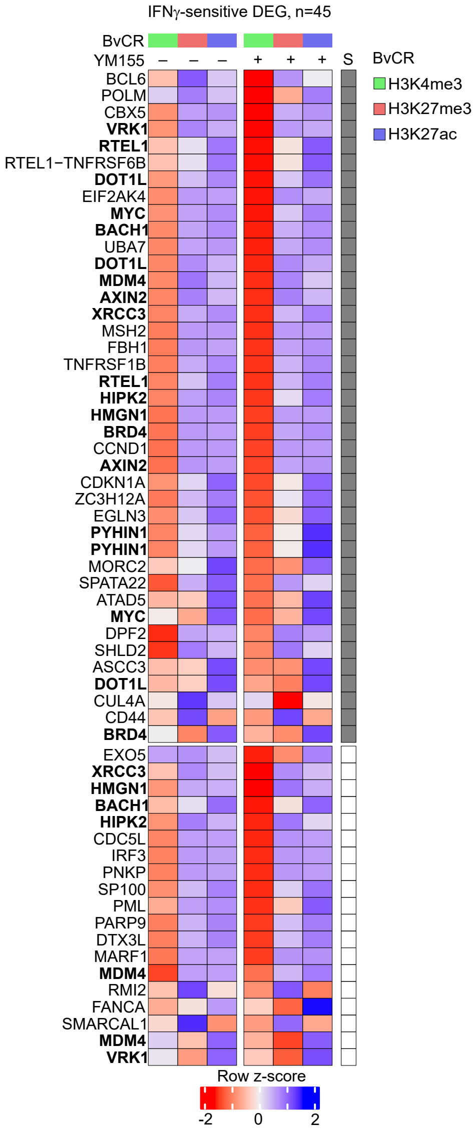

S4C. Heatmap of transcription change of DEG annotated to DNA Damage Response (DDR) pathway. Asterisks indicate RNAseq nominal p-values - * < 0.05, ** < 0.01, *** <0.001.

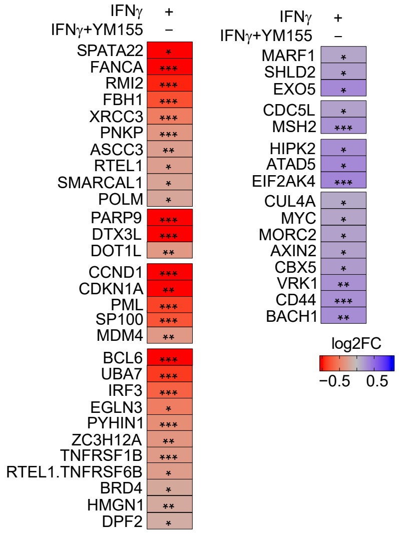

S4D. Box plot of quantified tag deposition in survivin-sensitive and IFNg-sensitive genes annotated to DNA damage response and connected to H3K4me3-BvCR. Mann-Whitney p-values are indicated

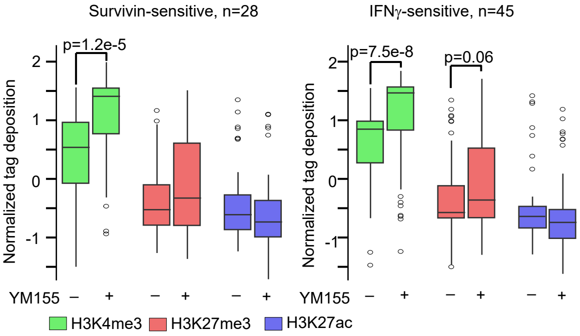

S5. Genomic maps of the DNA repair gene loci MSH6, FANCI, SMC3, PIAS4, and MRE11. Filled black and red boxes indicate cis-RE connected to the gene, as determined by GeneHancer. Distance to TSSs is shown. Black filled peaks underneath cis-RE indicate the positions of survivin-ChIP and histone H3-ChIP peaks. Colored peaks indicate the change in tag deposition for H3K4me3 (green) and H3K27me3 (red) after YM155 treatment, scaled to enable direct comparison between the two modifications.

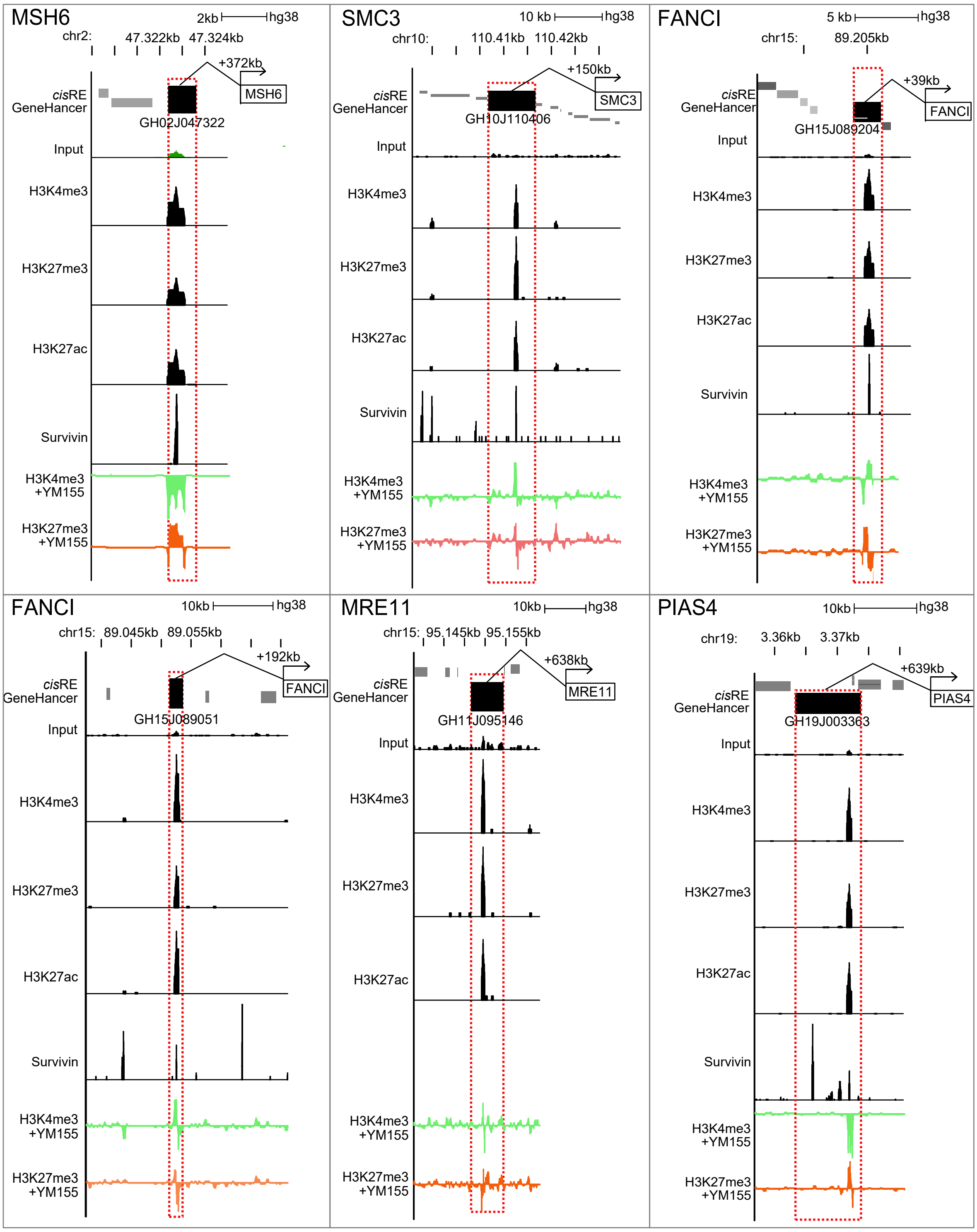

S6A. Frequency of overlapping BvCR with cBAF and PBAF complex subunits retrieved from ReMap2022 database. Fisher test p-values are indicated. (right) Table of BRG1/SWI proteins identified by mass spectrometry. Enrichment score, number of unique peptides, and sequence coverage are calculated by MaxQuant software.

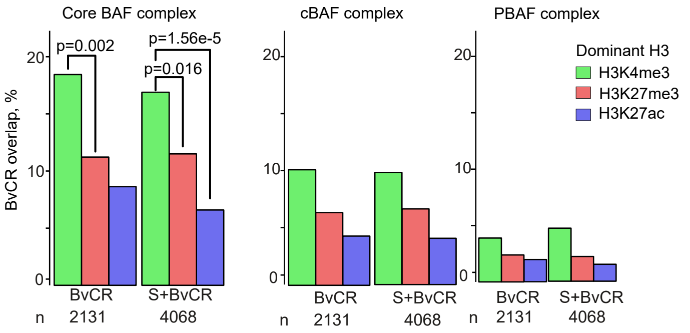

S6B. Coomassie-stained electrophoresis gel depicts replicate nuclear extracts of input (1anes 2 and 3), survivin-IP (lanes 7 and 10) and non-specific IgG IP (lanes 15 and 16). The red-marked bands were excised for interrogation using LC-MS. Molecular weight ladder (MW) is shown on the left side and in lane 14. Experiment 1 is presented in lanes 2, 7 and 15; experiment 2 is presented in lanes 3, 10 and 16. Red numbers indicate the bands analyzed by mass spectrometry

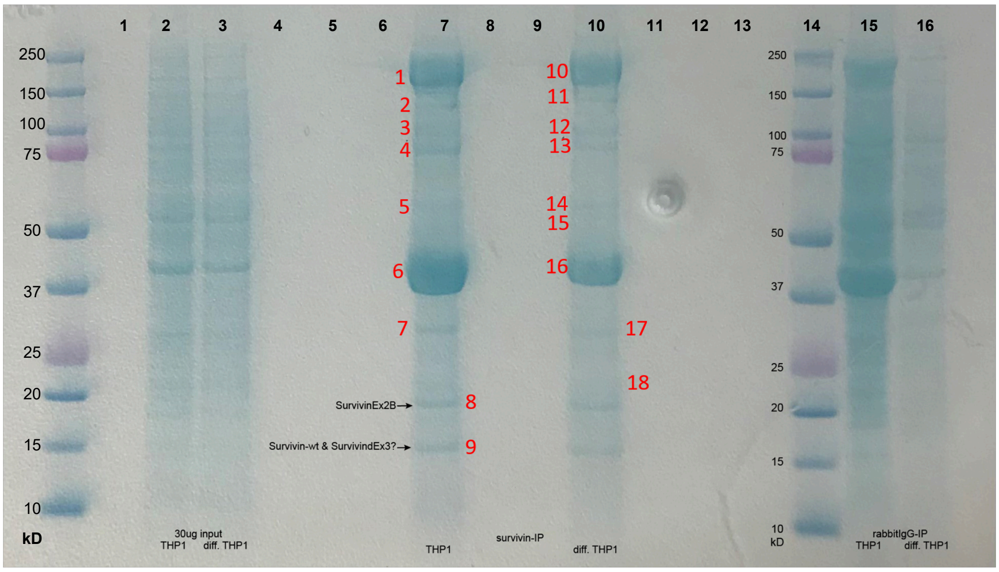

S6C. Distribution of survivin binding probability across the protein sequence of SMARCC2, SMARCD1, and SMARCE1. $M_{bind}(n)$ value indicate the fraction of mutations compatible with a survivin binding to the residue, defined by the functional composition of atomic group (Anindya et al, 2024). “1” indicates a region predicted to bind survivin even if the position is mutated to any other amino acid. “0” indicates no mutation can convert the site to survivin binding region. Uniprot IDs of the proteins are indicated in brackets.

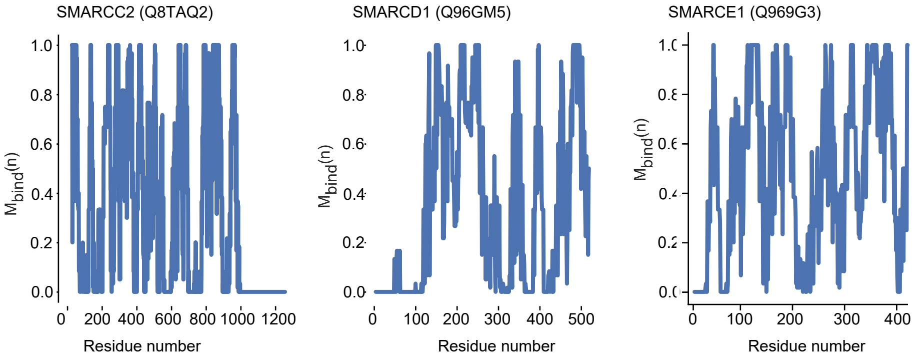

S7. Aminoacid residues of the interactions between survivin and subunits of the canonical BRG1/SWI complexes (S7A) and polybromo-BRG1/SWI complex (S7B), obtained through protein-protein docking and through peptide binding microarray.

S7A. Canonical BRG1/SWI

Conformation 1: Docking energy -354.10 kcal/mol

Subunits	Docking Residues of subunit to survivin	Peptide binding
SMARCA4	380-HIS, 381-ARG, 384-GLU, 396-LEU, 399-LYS, 403-GLU	No match
SMARCC2	600-GLU,	No match
	643-PRO, 646-ASP, 647-PRO, 650-GLU, 651-ASP, 656-LEU,	^{634}ECILHFLRL**P**IE**DP**YL**ED**SE^{653}
	682-SER, 683-VAL, 700-PHE, 701-SER, 702-LYS, 703-MET	No match
SMARCD1	480-GLU, 483-PHE, 485-PRO	^{469}VVGNPEEERRA**E** FY**F**^{483}
DPF2	65-GLY, 66-LEU, 67-ALA, 68-SER	No match
ARID1A	1722-ARG, 1726-GLU, 1732-LYS, 1738-ASP,	^{1709}LPGLLELLVEYF R**R**CLI**E**IFGI L**K**EYEVG**D**^{1738}
	1833-ARG,	^{1824}VVDCSDK LG**R**VQEFD^{1838}
	1853-GLU, 1855-ILE, 1862-LYS	No match

Conformation 2: Docking energy -318.61 kcal/mol

Subunits	Docking Residues of subunit to survivin	Peptide binding
SMARCA4	452-ILE, 455-LYS, 456-LEU, 459-GLN, 460-GLN	No match
ACTL6A	339-ASP	No match
SMARCB1	217-MET	^{214}TPE**M**FSEILCDDLDLNPLTF^{233}
SMARCD1	353-LYS, 355-SER, 359-GLN	No match
SMARCE1	172-SER, 173-ILE	^{168}PYM**SI**QPAEDPDDYDDGFSM^{188}
ARID1A	1666-THR, 1667-PRO, 1668-GLU, 1669-ALA, 1670-TRP	No match
	1699-ASN, 1702-MET, 1703-THR, 1705-ASN, 1707-SER, 1708-GLN	^{1684}STWALDTINILLYDD**N**SI**MT**^{1703}
	1994-VAL, 1995-PRO, 1996-GLY, 2228-VAL	No match
	2265-PRO, 2267-MET, 2268-ASN, 2269-SER	^{2249}VDENHSEFTLYESRLL**DISV**^{2268}

Conformation 3: Docking energy -330.56 kcal/mol

Subunits	Docking Residues of subunit to survivin	Peptide binding
SMARCA4	467-LYS, 479-ILE, 482-HIS, 486-PHE	No match
ACTL6A	402-SER, 404-GLY, 407-GLN, 408-GLN	^{404}**G**TF**QQ**MW ISKQEYEE^{418}
SMARCB1	7-SER, 8-LYS,	No match
	38-MET, 46-ARG, 47-TYR, 48-PRO, 49-SER	No match
	98-GLU, 99-ILE, 100-LEU, 101-ASP	No match
	183-PRO	
	209-ASN, 211-LYS, 214-THR	^{214}**T**PEMFSEILCDDLDLNPLTF^{233}
	242-ARG, 245-ILE, 246-GLU, 247-SER, 248-TYR, 249-PRO	
	323-GLN, 324-LYS, 380-ASN	No match
ARID1A	1845-ARG	No match
	2074-SER, 2075-PRO, 2076-TYR, 2077-PRO	^{2059}LANISGQLDL**SPYP**E^{2078}

S7B. Polybromo BRG1/SWI

Conformation 1: Docking energy -377.20 kcal/mol

Subunits	Docking Residues of subunit to survivin	Peptide binding
SMARCA4	437-LYS, 438-ALA	^{429}ALETALNA**KA**YKRSK^{443}
SMARCC2	614-GLU, 615-MET, 616-TYR, 617-LYS, 618-ASP	No match
SMARCD1	505-GLU, 508-GLN, 509-ALA, 510-LEU	No match
PHF10	280-ASN, 281-THR	No match
ARID2	187-SER, 188-LYS	unknown
BRD7	378-LEU, 379-GLN, 380-SER	unknown
PBRM1	1599-PRO, 1601-THR, 1604-LEU	unknown

Conformation 2: Docking energy -371.23 kcal/mol

Subunits	Docking Residues of subunit to survivin	Peptide binding
SMARCA4	468-ARG, 472-HIS, 476-LEU	No match
SMARCC2	925-GLN, 926-GLN, 929-ALA, 930-ASP, 931-ARG, 932-GLN, 933-ALA, 934-PHE, 935-HIS, 936-MET, 937-GLU, 938-GLN, 941-TYR, 944-MET	No match
SMARCE1	274-CYS, 275-GLY, 276-LEU	No match
SMARCD1	325-THR, 326-HIS, 327-LYS, 331-PRO, 332-HIS, 334-ARG	No match
ARID2	1816-TYR, 1820-PHE, 1821-THR	No information
ARP4	405-THR, 407-GLN, 408-GLN, 409-MET, 410-TRP, 411-ILE, 412-SER, 413-LYS, 414-GLN, 415-GLU, 416-TYR	No information
	1599-PRO, 1601-THR, 1604-LEU	unknown

Conformation 3: Docking energy -369.97 kcal/mol

Subunits	Docking Residues of subunit to survivin	Peptide binding
SMARCA4	362-ASP, 364-VAL, 365-GLU, 366-ILE, 368-GLN, 369-GLU, 371-GLU, 372-TYR, 383-GLN, 386-GLU	No match
SMARCE1	185-GLY, 186-PHE, 190-HIS	^{168}PYMSIQPAEDPDDYDD GFSMKH^{190}
SMARCD1	126-LYS	No match
ARID2	187-SER, 188-LYS	No information
BRD7	380-SER, 386-GLN, 388-PHE, 389-LYS, 433-TYR, 558-GLU, 561-ASN, 562-GLU, 563-ARG, 565-SER, 566-THR, 567-ARG, 568-PRO, 569-PRO, 570-PRO, 571-ASN, 572-MET, 573-ILE, 575-LEU, 576-LEU, 577-GLY, 578-PRO, 579-SER, 580-TYR	No information
PBRM1	1590-THR, 1591-THR, 1599-PRO, 1643-GLU, 1644-GLN, 1645-GLU, 1647-ARG, 1648-LEU, 1649-PRO, 1650-SER, 1651-HIS	No information
	1599-PRO, 1601-THR, 1604-LEU	unknown

S8. Gallery of immunohistochemical images depicting colocalization of survivin (red) and BRG1 (yellow) in nucleus (blue) of THP1 cells, visualized by confocal microscopy at resolution 40X. Nuclear area is identified by Hoechst stain. Overlap coefficient was calculated by colocalization of fluorescence pixels using ImageJ JACoP plugin

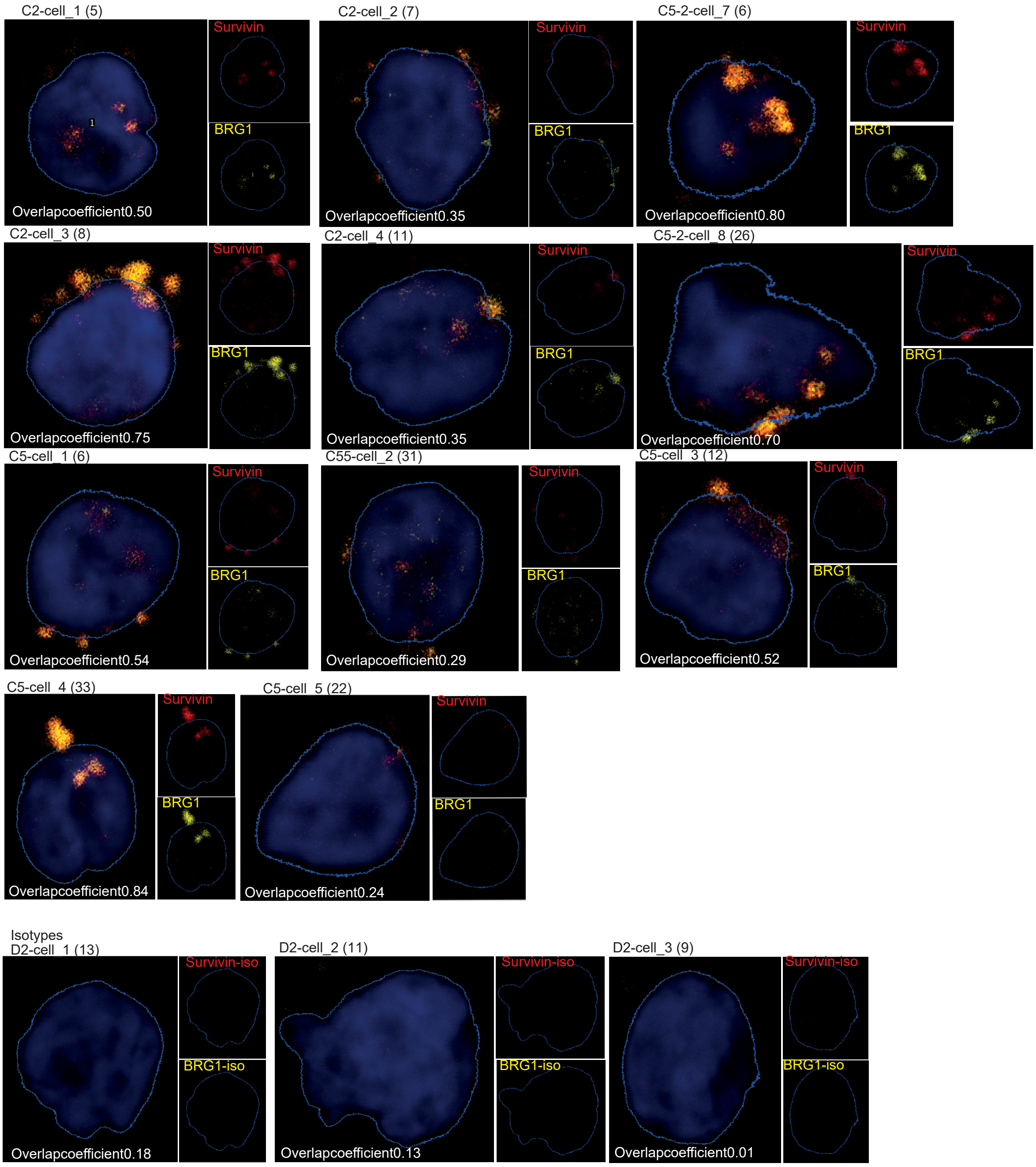

S9. DNA Damage Response (DDR) network map of upregulated (red) and downregulated (blue) differentially expressed genes in $BRG1^{hi}$ cells in patients with rheumatoid arthritis. Nodes are colored by fold expression difference (log2FC) between $BRG1^{hi}$ and $BRG1^{lo}$ $CD4^{+}$ cells of genes within nodes. Size of bubble corresponds to percentage of $BRG1^{hi}$ genes within each node.

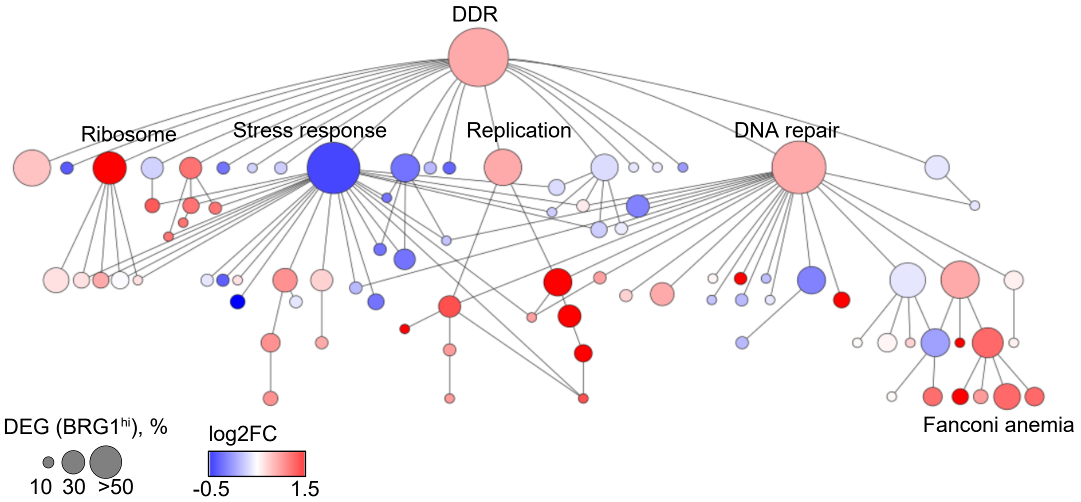

Supplement: Supplementary file 1 — Additional file 1: Supporting Figure S1. S1A. The table of clinical characteristics of healthy controls and patient material used in this study. S1B. The table of primers used for qPCR analysis in this study. S2A. Box plots of histone H3 tag deposition within the bivalent chromatin regions (BvCR) dominant by H3K4me3, H3K27me3 and H3K27ac. S2B. Box plots of histone peak scores within BvCR dominant by H3K4me3, H3K27me3 and H3K27ac. Kolmogorov–Smirnov test p-values are shown. S2C. Box plot of percentage tag change for histone peaks within H3K27me3- and H3K27ac-BvCR, after YM155 treatment. Mann–Whitney test p-values are indicated. Supporting Figure S3. S3A. Forest plot of probability of BvCR to be changeable (Ch) in YM155-treated CD4 cells and genes connected to BvCR to be differentially expressed (DEG) in CD4 + cells treated with IFNγ or IFNγ + YM155. S3B. Scatter plot of correlation between change in deposition of H3K4me3 and H3K27me3 tags and change in transcription of DEG after treatment with IFNγ or IFNγ + YM155 in survivin-positive H3K4me3-BvCR. Spearman ρ are indicated. S3C. Radar plot of Spearman’s ρ correlations between tag change in H3K4me3 and H3K27me3 deposition in H3K27me3-BvCR and transcription change of DEG in CD4+ cells treated with IFNγ or IFNγ + YM155. Arrows indicate direction of transcription change. Supporting Figure S4. S4A. Bar plot of frequency of genes connected to BvCR in the enriched pathways. To the right is the Venn diagram of IFNγ- and survivin-sensitive genes connected to H3K4me3-BvCR and annotated to the DNA damage response pathway (GO:0006974). S4B. Heatmap of normalized tag deposition in BvCR connected to DEG treated with IFNγ + YM155. Filled squares indicate colocalization of survivin (S) in the BvCR. Genes connected to multiple BvCR are marked in bold. S4C. Heatmap of transcription change of DEG annotated to DNA Damage Response (DDR) pathway. Asterisks indicate RNAseq nominal p-values—* < 0.05, ** < 0.01, *** < 0.001. S4D. Box plot of [file 12964_2024_1814_MOESM1_ESM.pdf]
